# Supplementary material for: Reversible single crystal photochemistry and spin state switching in a metal-cyanide complex
Source: Nat Commun. 2025 Sep 29;16:8377. doi: 10.1038/s41467-025-63523-x (PMC12480581; doi:10.1038/s41467-025-63523-x)
Supplement: Supplementary file 1 — Supplementary Information [file 41467_2025_63523_MOESM1_ESM.pdf]

Supplementary Information

Reversible single crystal photochemistry and spin state switching in a metal-cyanide complex

Michał Magott, Mirosław Arczyński, Leszek Malec, Michał Rams, Mathieu Rouzières, Andrei Rogalev, Fabrice Wilhelm, Itziar Oyarzabal, Thomas Lohmiller, Alexander Schnegg, Coen de Graaf, Corine Mathonière, Rodolphe Clérac, and Dawid Pinkowicz*

*corresponding author email address: dawid.pinkowicz@uj.edu.pl

List of items in the SI:

Figure S1. Experimental powder X-ray diffraction pattern obtained for $K_4[Mo^{III}(CN)_7]\cdot 2H_2O$ (**1**) at 296(2) K (black line) and the calculated pattern for the single-crystal structure at 90(2) K (grey line) reported by Ohkoshi et al.[1]

Figure S2. a, Solid state infrared spectrum of **1** recorded at room temperature and **b**, magnification of the 2300-1850 cm^{-1} region of the spectrum showing the cyanide stretching band.

Additional single-crystal X-ray diffraction details

Table S1. Details of crystal structure measurements and structural refinements.

Table S2. Continuous shape measure (CShM) analysis[2] for Mo coordination spheres in the analyzed scXRD structural models. Note that values closer to 0 indicate better agreement with the reference geometry: PBPY-7: pentagonal bipyramid; COC-7: capped octahedron; CTPR-7: capped trigonal prism; PPY-6: pentagonal pyramid; OC-6: octahedron; TPR-6: trigonal prism.

Figure S3. Crystal packing diagrams for 1 and 2. Crystal packing for **1** before irradiation is shown in panels A1 and A2 while for **2** after 405 nm irradiation is presented in panels B1 and B2. The panels on the left show nine neighboring unit cells along the *b* crystallographic axes while selected crystal structure fragments where the cyanide dissociation occurs is shown on the right. A1 and B1 show the dissociation of the Mo-C bonds and the change of CN···K interatomic contacts upon light irradiation (H_2O molecules are omitted for clarity here): after the Mo-C bond photodissociation the C7N7 cyanide is effectively stabilized between two potassium ions with the shortest K···C distances of 2.769 and 2.895 Å. A2 and B2 demonstrate the stabilizing role of the H-bonds which are formed upon 405 nm irradiation between C7N7 and the water molecules designated by the positions of O1 and O2.

Figure S4. Mo-C bond length analysis in **1**, **2**, $Li_3[Mo^{III}(CN)_6]\cdot 6DMF$ and $[K(crypt\text{-}222)]_3[Mo^{III}(CN)_6]\cdot 2CH_3CN$ (**3**).

Figure S5. Schematic representation of the photochemical transformation from **1** to **3** in acetonitrile solution at room temperature.

Figure S6. UV-vis spectra for 0.5 mM solutions of **1** in CH_3CN (solubilized with four equivalents of crypt-222) recorded at room temperature before irradiation (green line) and after irradiation (blue line): **a**, after 20 minutes of violet light irradiation (λ = 420-430 nm) and **b**, 5 minutes of white LED light irradiation.

Description of the crystal structure of [K(crypt-222)]$_3$[Mo^{III}(CN)$_6$]·2CH$_3$CN (**3**)

Figure S7. Asymmetric unit of [K(crypt-222)]$_3$[Mo^{III}(CN)$_6$]·2CH$_3$CN (**3**) at 100 K. Ellipsoids are depicted at 50% probability level.

Table S3. Crystal structure parameters for [K(crypt-222)]$_3$[Mo^{III}(CN)$_6$]·2CH$_3$CN (**3**).

Table S4. Selected bond lengths and angles in the structure of [K(crypt-222)]$_3$[Mo^{III}(CN)$_6$]·2CH$_3$CN (**3**) at 100 K.

Figure S8. Experimental powder X-ray diffraction pattern recorded for **3** at 296(2) K (black line) and calculated pattern from the single crystal structure studied at 293(2) K (gray line).

Figure S9. Comparison of the UV-vis spectra of **3** in the solid state (black line) and as a 0.5 mM solution in anhydrous acetonitrile (blue line) at room temperature.

Figure S10. Selected optical reflectivity spectra for **1** between 400 and 1000 nm at selected temperature between 295 and 10 K recorded in the dark and at a scan rate of 4 K min^{-1} in **a**, cooling mode and **b**, heating mode. **c**, Variation of the absolute optical reflectivity (ΔAR) recorded at 550 nm for **1** comparing before and after excitation with different LEDs ($\Delta AR = AR_{after} - AR_{before}$; 10 minutes, at 2 mW cm^{-2}) at 10 K (after a fast cooling of the sample from room temperature in the dark). **d**, Optical reflectivity spectra for **1** at 10 K before and after excitation with a 405-nm LED (10 minutes, at 2 mW·cm^{-2}). **e**, Selected optical reflectivity spectra for **1** at 10 K recorded during a 2-hours irradiation with 405-nm LED (at 5 mW·cm^{-2}). In insert, time evolution of the 550-nm *AR* signal during 405-nm irradiation (10 K; 5 mW·cm^{-2}). **f**, Thermal variation of the 550-nm reflectivity signal in the dark (in blue, almost temperature independent around 0.2), during irradiation at 10 K with 405 nm LED (2 mW·cm^{-2}; in violet), and after irradiation in the dark (in red). A spectroscopic white light of 0.08 mW·cm^{-2} was used for these measurements.

Figure S11. **a**, Variation of the absolute optical reflectivity (ΔAR) for **1** recorded at 550 nm and at 10 K (after a fast cooling of the sample from room temperature in the dark and a 385-nm excitation during 10 minutes at 2 mW·cm^{-2}) comparing before and after desexcitation with different LEDs ($\Delta AR = AR_{after} - AR_{before}$; 10 minutes, at 10 mW cm^{-2}). **b**, Time evolution of the 550-nm *AR* signal during four irradiation cycles of 385-nm excitation (10 K; 1 hour, 5 mW·cm^{-2}; in violet) and 660-nm desexcitation (10 K; 2 hours, 10 mW·cm^{-2}; in red). **c**, Selected optical reflectivity spectra for **1** at 10 K recorded in the dark (blue trace), after one 385-nm irradiation (10 K; 1 hour, 5 mW·cm^{-2}; violet trace) and a subsequent 660-nm irradiation (10 K; 2 hours, 10 mW·cm^{-2}; red trace). A spectroscopic white light of 0.08 mW·cm^{-2} was used for these measurements.

Figure S12. a, IR spectrum of **3** recorded in the solid state at room temperature and **b**, close-up of the cyanide stretching region.

Figure S13. Mo L_3-edge XANES spectra at room temperature of **1** in the solid state (solid line) and in water solution (dashed line) (**a**) and **3** in the solid state (solid line) and in the acetonitrile solution (dashed line).

Table S5. Positions and assignments of all excitations from theoretical calculations using CASSCF/CASPT2 (complete active space self consistent field/complete active space perturbation theory) approaches for metal-centered states of **1**.[a]

Table S6. Positions and assignments of all excitations from theoretical calculations using CASSCF/CASPT2 approaches for metal-centered states of **2**.[a]

Details of spin-polarized periodic DFT computations

Table S7. Relative errors in reproducing experimental unit cell volumes ($\mathbf{1}_{\mathbf{test}}$ for Δ*V*[**1**] and Δ*V*[**2**⟶**1**], **2** for Δ*V*[**1**⟶**2**] and Δ*V*[**2**]) for studied DFT functionals.

Figure S14. Comparison of Mo···CN distances (for the dissociated CN^- ligand) obtained by geometry optimization of structures **2** and **1**⟶**2** for studied DFT functionals. Please note that in most cases the circles and squares overlap.

Table S8. Comparison of atomic charges at Mo centers ($Z_{MUL(\alpha-\beta)}$ in a.u.) obtained in the Mulliken population analysis of α-β electrons for studied DFT functionals.

Figure S15. Figure of Merit (defined as described above) obtained by geometry optimization of structures **1**⟶**2** and **2**⟶**1** for studied DFT functionals.

Figure S16. Mo···CN distances (blue; right axis) and unit cell volumes (black; left axis) for the optimized geometries of **1**, **1**⟶**2**, **2** and **2**⟶**1** for HISS functional.

Figure S17. Overlay of the crystal structures: **a**, experimental **2** (black) and optimized **1**⟶**2** (blue) and **b**, experimental **1** (black) and optimized **2**⟶**1** (red), for HISS functional.

Description of the bulk magnetic properties of $K_4[Mo^{III}(CN)_7]\cdot 2H_2O$ (1) in the ground state

Figure S18. a, Temperature (*T*) dependence of the χT product at 0.1 T for **1** (where $\chi = M/H$ is the molar magnetic susceptibility normalized per complex) and **b**, field (*H*) dependence of the magnetization (*M*) for **1** at 1.8 K.

Figure S19. a, Temperature (*T*) dependence of the molar magnetic susceptibility, χ, at 0.1 T for **1** (black points) and fit to Bonner-Fisher model for a uniform chain of antiferromagnetically coupled S = 1/2 Heisenberg spins (green line; *vide supra*). **b**, Temperature dependence of the heat capacity, $C_p(T)$, for **1** under zero magnetic field (open circles), Debye model used to determine the nonmagnetic phonon contribution to the heat capacity ($C_{background}$, red line), and the magnetic contribution to the heat capacity, C_{pm}, defined as C_p-$C_{background}$ (full circles).

Figure S20. Fragments of the crystal structures at 30 K of **1** (a) and **2** (b) highlighting the supramolecular chain arrangement in **1**, and its disappeareance in **2** due to the cyanide dissociation.

Description of the bulk magnetic properties of [K(crypt-222)]$_3$[Mo^{III}(CN)$_6$]·2CH$_3$CN (3)

Figure S21. Temperature (*T*) dependence of the χT product at 0.1 T for **3** (where $\chi = M/H$ is the molar magnetic susceptibility normalized per complex). The solid purple line is the best fit of the data to a *S* = 3/2 Curie law (with g_{Mo} being the only ajustable parameter).

Figure S22. Field (*H*) dependence of the magnetization (*M*) for **3** at 2.0 K. The solid purple line is the simulated *S* = 3/2 Brillouin function with g_{Mo} =1.97.

Table S9. Stretched exponential decay fits of the relaxation data for **2** shown in Fig. 3e.[a]

Figure S23. Photomagnetic studies of 1 after irradiation at 100 K. a, Time evolution of the χT product (at 0.1 T) of **1** during 4 consecutive cycles of 405 nm irradiation at 100 K, each followed by thermal relaxation at 220 K, confirming reversible photo-induced transformation **1**→**2**; **b**, Temperature dependence of the χT product at 0.1 T before (**1**) and after 405 nm irradiation (**2**) at 100 K in four consecutive irradiation and heating cycles as shown in Fig. S22a; **c**, Magnetization (*M*) versus magnetic field (*H*) plots for **1** (before irradiation) and **2** (after 405 nm irradiation) recorded at 2.0 K after four consecutive irradiation and heating cycles as shown in Fig. S22a. Dashed lines in all three plots indicate the χT (**a** and **b**) or M (**c**) values reached after the analogous irradiation experiments performed at 10 K presented in Figure 3.

Figure S24. Thermogravimetric (TG) analysis of K$_4$[Mo^{III}(CN)$_7$]·2H$_2$O (**1**) showing the complete loss of two water molecules of crystallization at 80 °C in a single step corresponding to 7.6% of the formula weight of **1** (*FW* = 470.5 g·mol^{-1}). Data collected under dry N$_2$ flow with 2 K·min^{-1} heating rate.

Figure S25. ORTEP-style illustration of the asymmetric unit of **1** (CCDC no. 2352257). Mo – green, K – violet, C – gray, N – blue, O – red. Ellipsoids are depicted at 50% probability level.

Figure S26. ORTEP-style illustration of the asymmetric unit of **2** (CCDC no. 2352263). Mo – green, K – violet, C – gray, N – blue, O – red. Ellipsoids are depicted at 50% probability level.

Figure S27. ORTEP-style illustration of the asymmetric unit of **1** relaxed by 638 nm irradiation (CCDC no. 2352259). Mo – green, K – violet, C – gray, N – blue, O – red. Ellipsoids are depicted at 50% probability level.

Figure S28. ORTEP-style illustration of the asymmetric unit of **1'** (CCDC no. 2352264). Mo – green, K – violet, C – gray, N – blue, O – red. Ellipsoids are depicted at 50% probability level.

Figure S29. ORTEP-style illustration of the asymmetric unit of **2'** (CCDC no. 2352261). Mo – green, K – violet, C – gray, N – blue, O – red. Ellipsoids are depicted at 50% probability level.

Figure S30. ORTEP-style illustration of the asymmetric unit of **1’** relaxed by heating to 200 K (CCDC no. 2352262). Mo – green, K – violet, C – gray, N – blue, O – red. Ellipsoids are depicted at 50% probability level.

Figure S31. ORTEP-style illustration of the asymmetric unit of $\mathbf{1_{test}}$ (CCDC no. 2352258). Mo – green, K – violet, C – gray, N – blue, O – red. Ellipsoids are depicted at 50% probability level.

Figure S32. ORTEP-style illustration of the asymmetric unit of $\mathbf{1_{test2}}$ (CCDC no. 2352260). Mo – green, K – violet, C – gray, N – blue, O – red. Ellipsoids are depicted at 50% probability level.

Figure S33. ORTEP-style illustration of the asymmetric unit of **3** at 100 K (CCDC no. 2352265). Mo – green, K – violet, C – gray, N – blue, O – red, H – white. Ellipsoids are depicted at 50% probability level.

Figure S34. ORTEP-style illustration of the asymmetric unit of **3** at 293 K (CCDC no. 2352266). Mo – green, K – violet, C – gray, N – blue, O – red, H – white. Ellipsoids are depicted at 50% probability level.

Note S1. List of all CheckCif A- and B-level alerts in the reported crystal structures with justifications (the same justifications were included in the cif files deposited with CSD).

Supplementary References

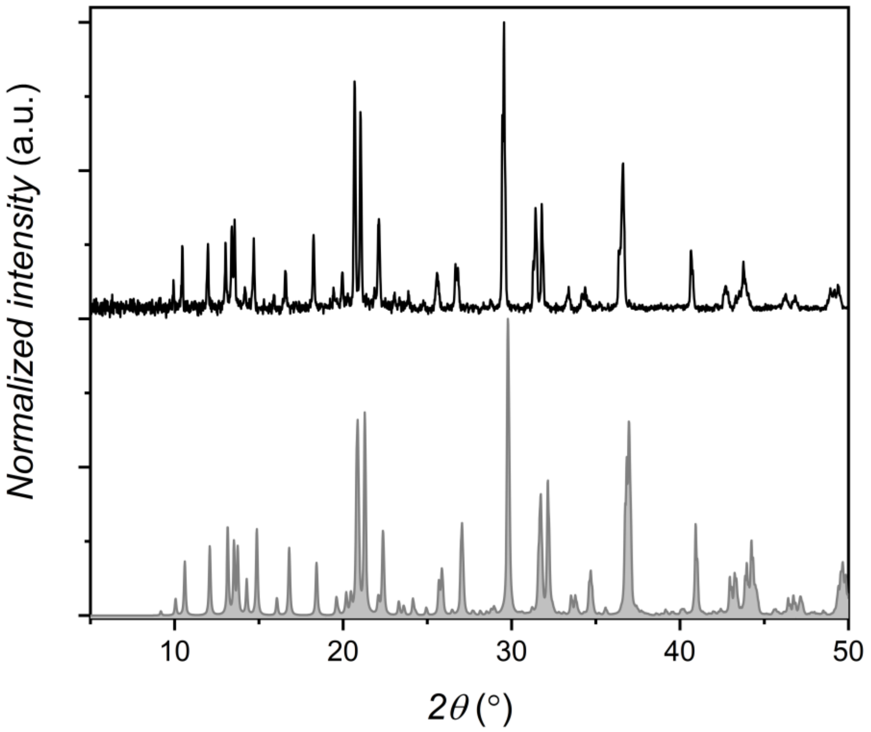

Figure S1. Experimental powder X-ray diffraction pattern obtained for $K_4[Mo^{III}(CN)_7]\cdot 2H_2O$ (**1**) at 296(2) K (black line) and the calculated pattern for the single-crystal structure at 90(2) K (grey line) reported by Ohkoshi et al.[1]

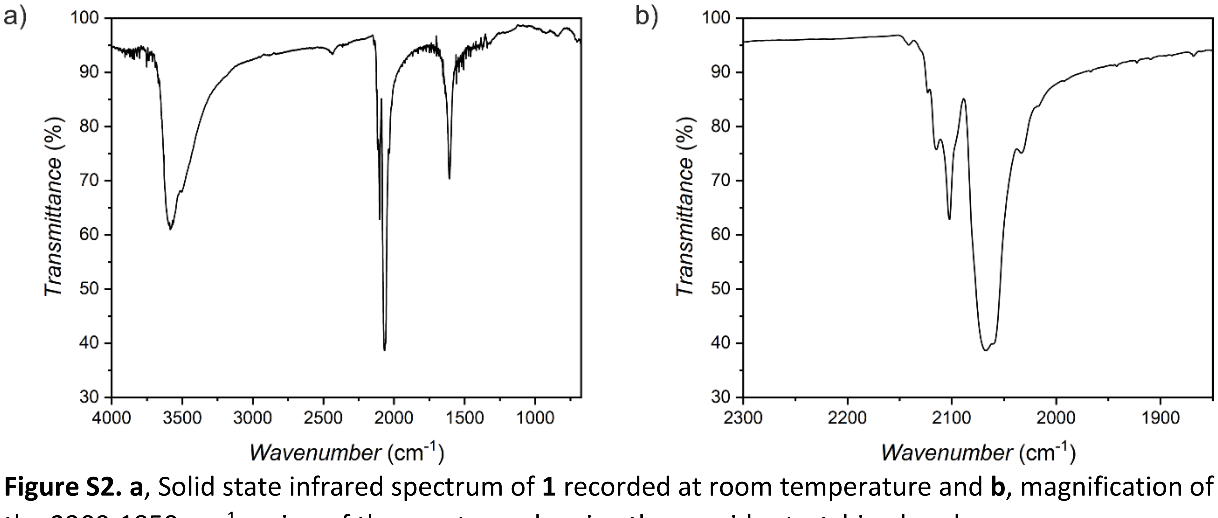

Figure S2. **a**, Solid state infrared spectrum of **1** recorded at room temperature and **b**, magnification of the 2300-1850 cm^{-1} region of the spectrum showing the cyanide stretching band.

Additional single-crystal X-ray diffraction details

In a preliminary experiment, the initial structure obtained for **1** using synchrotron radiation at 30 K showed a lightly larger unit cell volume of 757.28(9) Å^3 (Table S1, **1**$_{test}$) compared to 752.48(6) Å^3 recorded previously at 90 K using a commercial low-flux sealed-tube Mo X-ray source.[1] Repeated measurements on the same single crystal led to a further volume increase of 7 Å^3 (up to 764.27(16) Å^3, Table S1, **1**$_{test2}$) and an increase in electron density near the molybdenum ion. These changes were induced by X-ray radiation, and thus the X-ray flux and measurement time were optimized in subsequent experiments to afford the best possible structural parameters with minimal radiation damage.

Table S1. Details of crystal structure measurements and structural refinements.

compound		**1**$_{test}$	**1**$_{test2}$
CCDC no.		2352258	2352260
method		single-crystal XRD	single-crystal XRD
formula		$C_7N_7Mo_1K_4O_2H_2$	$C_7N_7Mo_1K_4O_2H_2$
formula weight (g·mol^{-1})		468.50	468.50
T (K)		30(1)	30(1)
λ (Å)		0.6889 (synchrotron)	0.6889 (synchrotron)
crystal system		triclinic	triclinic
space group		$P\bar{1}$	$P\bar{1}$
unit cell	***a*** **(Å)**	8.9201(5)	8.9957(10)
	b **(Å)**	9.3166(6)	9.3395(11)
	c **(Å)**	9.7444(7)	9.7432(12)
	α **(deg)**	86.900(6)	86.908(11)
	β **(deg)**	81.977(6)	82.149(11)
	γ **(deg)**	70.807(6)	70.480(10)
V (Å^3)		757.28(9)	764.27(16)
Z		2	2
calculated density (g·cm^{-3})		2.055	2.036
absorption coefficient (mm^{-1})		1.796	1.779
F(000)		454	454
θ range (deg)		2.244-28.987	2.243-29.071
collected reflections		14359	15513
R_{int}		0.0805	0.0926
completeness (%)		90.6	89.8
data/restraints/parameters		4004 / 3 / 208	4033 / 3 / 208
GOF on F^2		1.013	1.056
final R indices		R_1 = 0.0501 [$I > 2\sigma(I)$]<br>wR_2 = 0.1274 (all data)	R_1 =0.0783 [$I > 2\sigma(I)$]<br>wR_2 = 0.1960 (all data)
largest diff peak and hole		3.829 and -1.219 e·Å^{-3}	6.114 and -2.458 e·Å^{-3}
crystal size (mm x mm x mm)		0.05 x 0.03 x 0.01	0.05 x 0.03 x 0.01

Table S1(cont.). Details of the crystal structure measurements and structural refinements for the first crystal.

compound		**1**	**2**	**1 (relaxed by 638 nm irradiation)**
CCDC no.		2352257	2352263	2352259
method		single-crystal XRD	single-crystal XRD	single-crystal XRD
formula		$C_7N_7Mo_1K_4O_2$	$C_7N_7Mo_1K_4O_2$	$C_7N_7Mo_1K_4O_2$
formula weight (g·mol^{-1})		466.48	468.48	466.48
T (K)		30(1)	30(1)	30(1)
λ (Å)		0.6889 (synchrotron)	0.6889 (synchrotron)	0.6889 (synchrotron)
crystal system		triclinic	triclinic	triclinic
space group		$P\bar{1}$	$P\bar{1}$	$P\bar{1}$
unit cell	***a*** **(Å)**	8.9352(10)	9.598(4)	9.0224(6)
	b **(Å)**	9.2992(9)	9.710(2)	9.3760(5)
	c **(Å)**	9.7380(16)	9.752(3)	9.7105(9)
	α **(deg)**	86.993(12)	66.10(2)	87.576(6)
	β **(deg)**	82.116(13)	87.91(3)	82.500(7)
	γ **(deg)**	70.754(9)	88.84(3)	70.809(5)
V (Å^3)		756.67(17)	830.4(5)	769.16(10)
Z		2	2	2
calculated density (g·cm^{-3})		2.047	1.866	2.014
absorption coefficient (mm^{-1})		1.797	1.627	1.757
F(000)		450	450	450
θ range (deg)		2.046-28.907	2.058-25.496	2.229-29.077
collected reflections		15026	12741	14567
R_{int}		0.0924	0.1160	0.0881
completeness (%)		0.978	0.976	0.978
data/restraints/parameters		3975/0/200	3306/0/200	4066/24/200
GOF on F^2		1.055	0.995	1.047
final R indices		R_1 = 0.0579 [$I > 2\sigma(I)$] wR_2 = 0.1501 (all data)	R_1 = 0.0567 [$I > 2\sigma(I)$] wR_2 = 0.1446 (all data)	R_1 = 0.1087 [$I > 2\sigma(I)$] wR_2 = 0.2703 (all data)
largest diff peak and hole		4.812 and -1.398 e·Å^{-3}	1.974 and -1.128 e·Å^{-3}	8.236 and -2.888 e·Å^{-3}
crystal size (mm x mm x mm)		0.04 x 0.03 x 0.01	0.04 x 0.03 x 0.01	0.04 x 0.03 x 0.01

Table S1(cont.). Details of crystal structure measurements and structural refinements for the second crystal.

compound		**1′**	**2′**	**1′ (relaxed by heating to 200 K)**
CCDC no.		2352264	2352261	2352262
method		single-crystal XRD	single-crystal XRD	single-crystal XRD
formula		$C_7N_7Mo_1K_4O_2$	$C_7N_7Mo_1K_4O_2$	$C_7N_7Mo_1K_4O_2$
formula weight (g·mol^{-1})		466.48	466.48	466.48
T (K)		30(1)	30(1)	30(1)
λ (Å)		0.6889 (synchrotron)	0.6889 (synchrotron)	0.6889 (synchrotron)
crystal system		triclinic	triclinic	triclinic
space group		$P\bar{1}$	$P\bar{1}$	$P\bar{1}$
unit cell	***a* (Å)**	8.9443(8)	9.6002(9)	8.9697(7)
	***b* (Å)**	9.3385(8)	9.6496(11)	9.3535(6)
	***c* (Å)**	9.7258(11)	9.7097(8)	9.7124(8)
	**α (deg)**	86.983(9)	65.687(9)	87.317(7)
	**β (deg)**	82.127(9)	87.767(8)	82.254(7)
	**γ (deg)**	70.611(8)	88.774(10)	70.731(6)
V (Å^3)		759.04(13)	819.08(15)	762.18(10)
Z		2	2	2
calculated density (g·cm^{-3})		2.041	1.891	2.033
absorption coefficient (mm^{-1})		1.791	1.650	1.784
F(000)		450	450	450
θ range (deg)		2.359-27.338	2.232-27.339	2.051-27.340
collected reflections		14530	15780	14721
R_{int}		0.1065	0.1057	0.1026
completeness (%)		0.976	0.976	0.977
data/restraints/parameters		3635/6/200	3915/6/199	3655/12/200
GOF on F^2		1.060	1.032	1.071
final *R* indices		R_1 = 0.0671 [$I > 2\sigma(I)$] wR_2 = 0.1805 (all data)	R_1 = 0.0621 [$I > 2\sigma(I)$] wR_2 = 0.1679 (all data)	R_1 = 0.0796 [$I > 2\sigma(I)$] wR_2 = 0.2241 (all data)
largest diff peak and hole		5.131 and -1.495 e·Å^{-3}	3.640 and -0.966 e·Å^{-3}	6.744 and -1.040 e·Å^{-3}
crystal size (mm x mm x mm)		0.04 x 0.03 x 0.01	0.04 x 0.03 x 0.01	0.04 x 0.03 x 0.01

Table S2. Continuous shape measure (CShM) analysis[2] for Mo coordination spheres in the analyzed scXRD structural models. Note that values closer to 0 indicate better agreement with the reference geometry: PBPY-7: pentagonal bipyramid; COC-7: capped octahedron; CTPR-7: capped trigonal prism; PPY-6: pentagonal pyramid; OC-6: octahedron; TPR-6: trigonal prism.

	**$[Mo^{III}(CN)_7]^{4-}$**		
**Structure**	**PBPY-7**	**COC-7**	**CTPR-7**
**1**	5.814	1.534	0.250
**1 (relaxed by 638 nm irradiation)**	5.735	1.564	0.189
**1'**	5.729	1.509	0.253
**1' (relaxed by heating to 200 K)**	5.666	1.549	0.206
	**$[Mo^{III}(CN)_6]^{3-}$**		
**Structure**	**PPY-6**	**OC-6**	**TPR-6**
**2**	27.270	0.213	15.102
**2'**	27.313	0.202	15.236
**3**	28.558	0.075	15.496

PBPY-7: pentagonal bipyramid; COC-7: capped octahedron; CTPR-7: capped trigonal prism; PPY-6: pentagonal pyramid; OC-6: octahedron; TPR-6: trigonal prism

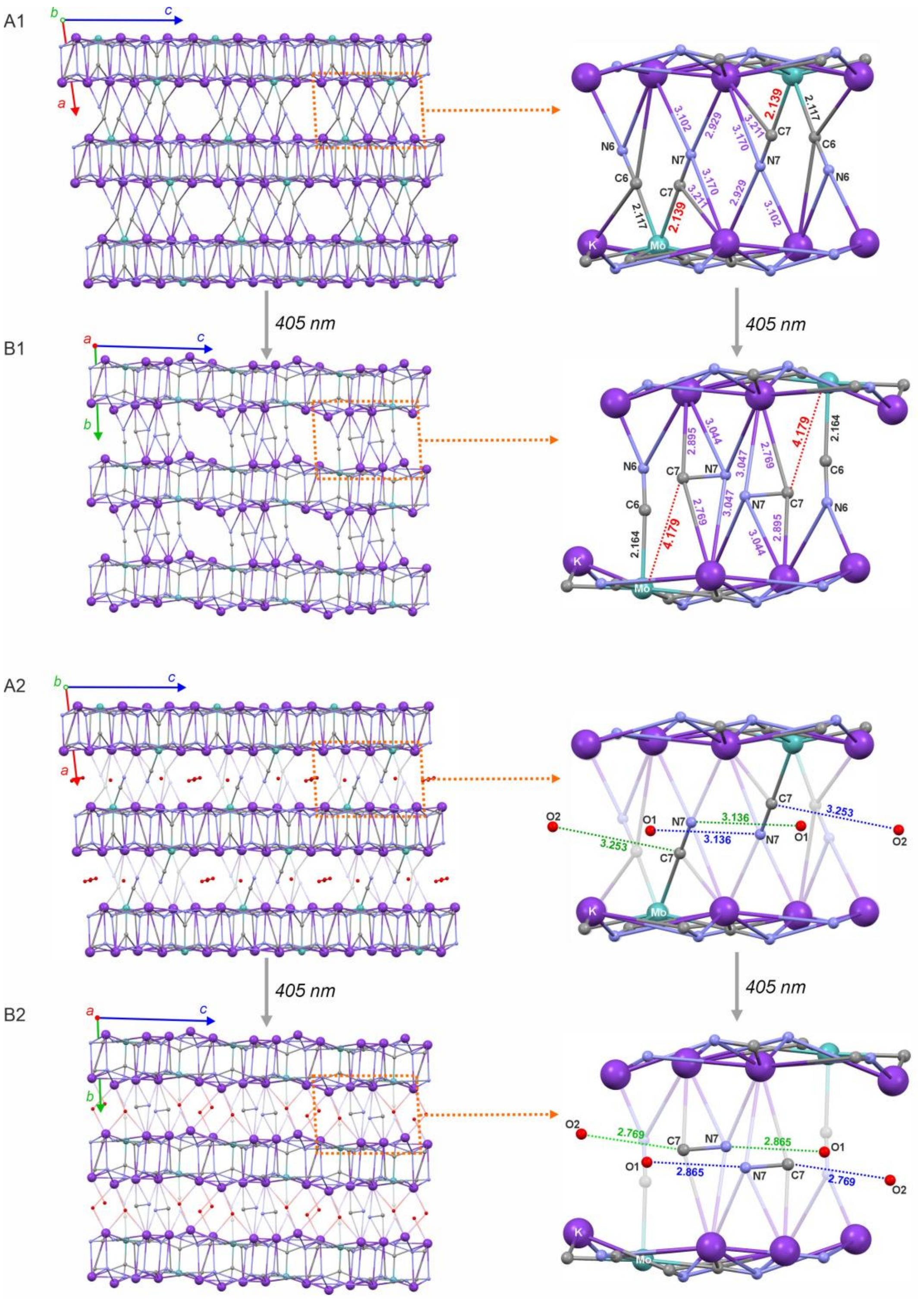

Figure S3. Crystal packing diagrams for 1 and 2. Crystal packing for **1** before irradiation is shown in panels A1 and A2 while for **2** after 405 nm irradiation it is presented in panels B1 and B2. The panels on the left show nine neighboring unit cells. The panels on the right visualize in detail the fragments where the cyanide dissociation occurs. A1 and B1 show the dissociation of the Mo-C bonds and the change of CN···K interatomic contacts upon light irradiation (H_2O molecules are omitted for clarity here): after the Mo-C bond photodissociation the C7N7 cyanide is effectively stabilized between two potassium ions with the shortest K···C distances of 2.769 and 2.895 Å. A2 and B2 demonstrate the stabilizing role of the H-bonds which are formed upon 405 nm irradiation between C7N7 and the water molecules designated by the positions of O1 and O2.

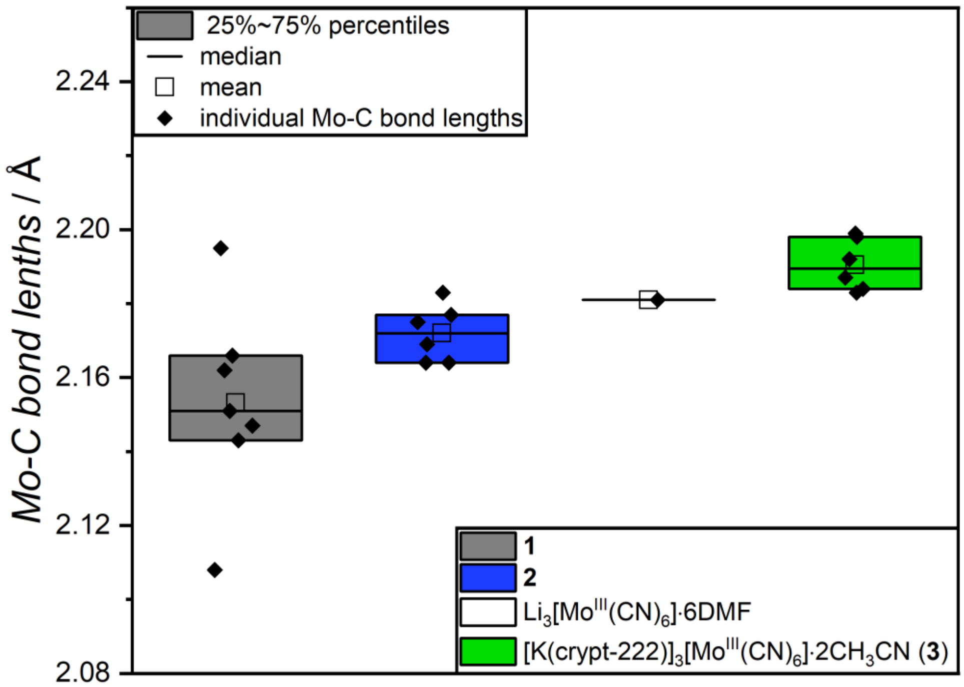

Figure S4. Mo-C bond length analysis in **1**, **2**, $Li_3[Mo^{III}(CN)_6]\cdot 6DMF$ [3] and $[K(crypt\text{-}222)]_3[Mo^{III}(CN)_6]\cdot 2CH_3CN$ (**3**).

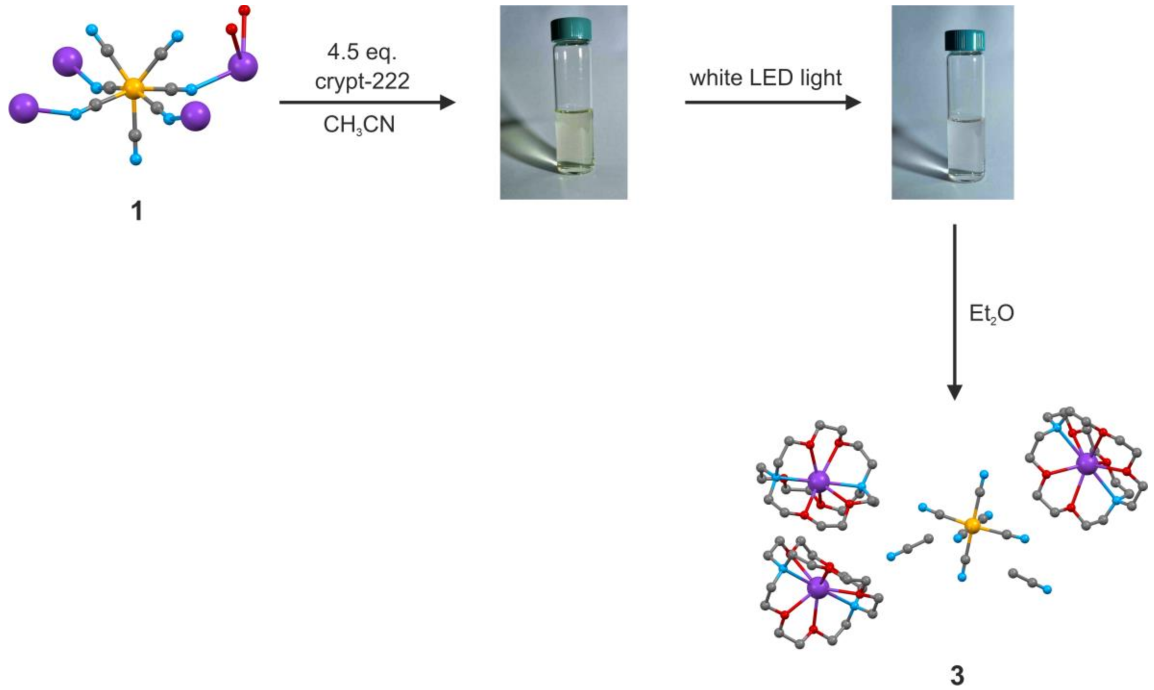

Figure S5. Schematic representation of the photochemical transformation from **1** to **3** in acetonitrile solution at room temperature.

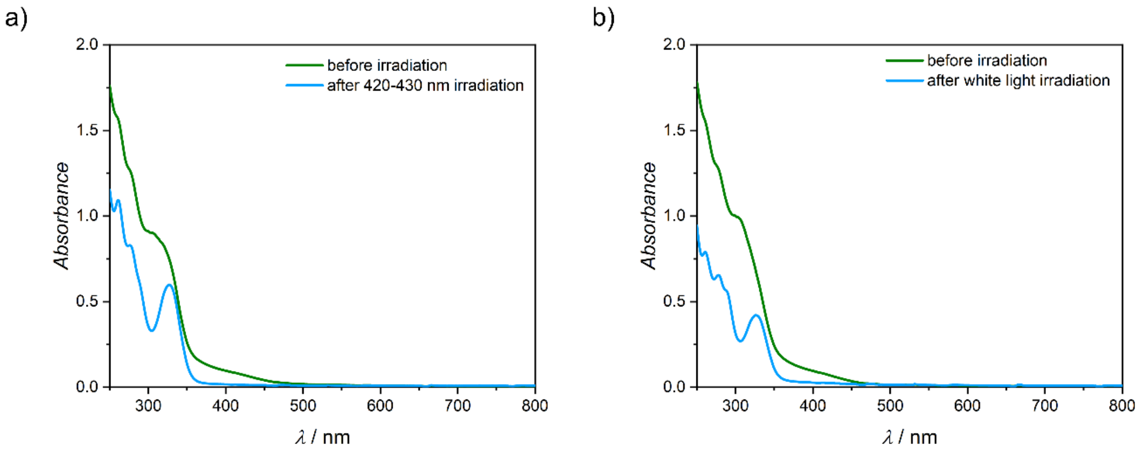

Figure S6. UV-vis spectra for 0.5 mM solutions of **1** in CH_3CN (solubilized with four equivalents of crypt-222) recorded at room temperature before irradiation (green line) and after irradiation (blue line): **a**, after 20 minutes of violet light irradiation (λ = 420-430 nm) and **b**, 5 minutes of white LED light irradiation.

Description of the crystal structure of [K(crypt-222)]$_3$[Mo^{III}(CN)$_6$]·2CH$_3$CN (**3**)

Compound **3** crystallizes in the orthorhombic *Pbca* space group, with the asymmetric unit corresponding to the empirical formula, [K(crypt-222)]$_3$[Mo^{III}(CN)$_6$]·2CH$_3$CN (Table S3). In its crystal structure, the [Mo^{III}(CN)$_6$]$^{3-}$ anion is surrounded by three [K(crypt-222)]$^+$ cations and two acetonitrile molecules as crystallization solvents (Fig. S7 – asymmetric unit). The hexacyanomolybdate(III) anion interacts through weak C-H···N contacts with methylene groups of [K(crypt-222)]$^+$ cations and with methyl groups of acetonitrile, effectively isolating each [Mo^{III}(CN)$_6$]$^{3-}$ unit. Similar to Li$_3$[Mo^{III}(CN)$_6$]·6DMF, the hexacyanomolybdate(III) anion in **3** exhibits nearly ideal octahedral geometry, as confirmed by continuous symmetry measures using the SHAPE software (Table S2).[2] The average Mo-C bond length in **3** is 2.191(6) Å at 100 K (Table S4), closely matching the 2.181(3) Å observed for the lithium salt at room temperature (Fig. S4). These structural parameters for [Mo^{III}(CN)$_6$]$^{3-}$ in **3** serve as a benchmark for comparison with the photoinduced state in **2**. Notably, the lengthening of the Mo-C bonds in **1** from 2.153(18) Å to 2.172(6) in **2** (and from 2.156(19) Å to 2.188(11) Å in the second crystal) aligns well with the 2.191(6) Å seen in **3**. This structural evidence supports the photoinduced tranformation scheme [Mo^{III}(CN)$_7$]$^{4-}$ → [Mo^{III}(CN)$_6$]$^{3-}$ + CN$^-$, further corroborated by spectroscopic and magnetic analyses.

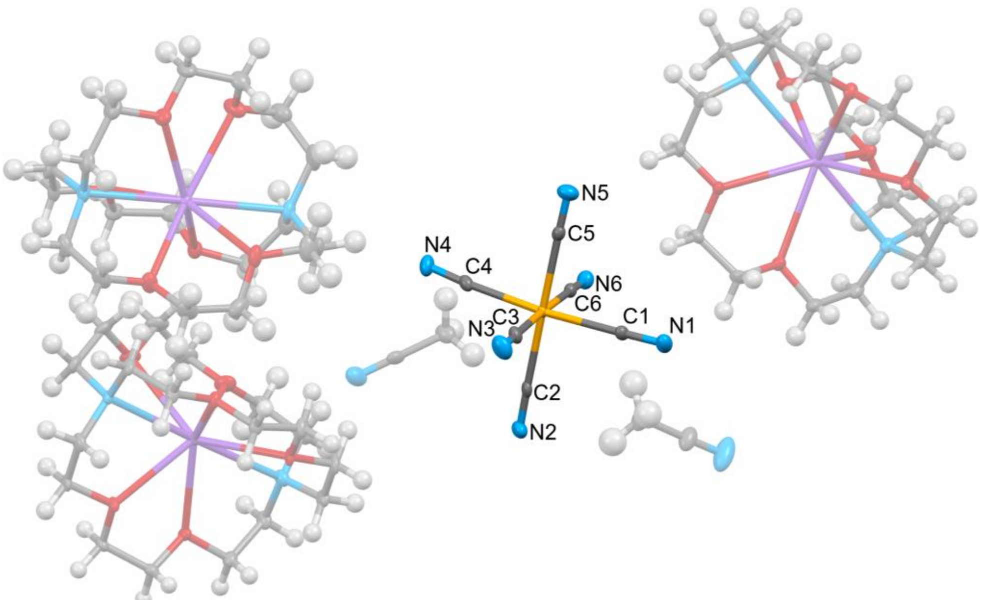

Figure S7. Asymmetric unit of [K(crypt-222)]$_3$[Mo^{III}(CN)$_6$]·2CH$_3$CN (**3**) at 100 K. Ellipsoids are depicted at 50% probability level.

Table S3. Crystal structure parameters for $[K(crypt\text{-}222)]_3[Mo^{III}(CN)_6]\cdot 2CH_3CN$ (**3**).

compound		**3** (100 K)	**3** (293 K)
CCDC no.		2352265	2352266
method		single-crystal XRD	single-crystal XRD
formula		$C_{64}H_{114}K_3MoN_{14}O_{18}$	$C_{64}H_{114}K_3MoN_{14}O_{18}$
formula weight (g·mol^{-1})		1580.93	1580.93
T (K)		100(2)	293(2)
λ (Å)		0.7107 (Mo Kα)	0.7107 (Mo Kα)
crystal system		orthorhombic	orthorhombic
space group		*Pbca*	*Pbca*
unit cell	***a* (Å)**	18.2778(5)	18.4282(14)
	***b* (Å)**	20.0943(6)	20.1869(18)
	***c* (Å)**	43.6202(11)	43.971(4)
V (Å^3)		16020.8(8)	16358(2)
Z		8	8
calculated density (g·cm^{-3})		1.311	1.284
absorption coefficient (mm^{-1})		0.39	0.38
F(000)		6712	6712
θ range (deg)		2.4-27.9	2.7-23.4
collected reflections		154424	150923
R_{int}		0.061	0.132
completeness (%)		99.8	98.4
data/restraints/parameters		19084 / 0 / 903	14756 / 70 / 903
GOF on F^2		1.20	1.05
final *R* indices		R_1 = 0.067 [$I > 2\sigma(I)$]<br>wR_2 = 0.124 (all data)	R_1 = 0.081 [$I > 2\sigma(I)$]<br>wR_2 = 0.187 (all data)
largest diff peak and hole		1.20 and -1.65 e·Å^{-3}	1.00 and -1.13 e·Å^{-3}
crystal size (mm x mm x mm)		0.40 x 0.30 x 0.16	0.36 x 0.26 x 0.10

Table S4. Selected bond lengths and angles in the structure of $[K(crypt\text{-}222)]_3[Mo^{III}(CN)_6]\cdot 2CH_3CN$ (**3**) at 100 K.

**Bonds / Å**		**Angles / °**	
Mo-C1	2.187(3)	Mo-C1-N1	177.3(3)
Mo-C2	2.199(3)	Mo-C2-N2	177.3(3)
Mo-C3	2.198(3)	Mo-C3-N3	179.0(3)
Mo-C4	2.183(3)	Mo-C4-N4	175.8(3)
Mo-C5	2.184(3)	Mo-C5-N5	179.0(3)
Mo-C6	2.192(3)	Mo-C6-N6	179.1(3)
Mo-C average	2.191(6)	Mo-C-N average	177.9(11)
**Angles / °**			
C1-Mo-C2	91.39(11)	C1-Mo-C4	174.39(11)
C1-Mo-C3	91.03(12)	C2-Mo-C5	177.99(11)
C1-Mo-C5	88.66(11)	C3-Mo-C6	178.23(12)
C1-Mo-C6	87.84(11)		
C2-Mo-C3	91.21(11)		
C2-Mo-C4	92.34(12)		
C2-Mo-C6	87.47(11)		
C3-Mo-C4	93.08(12)		
C3-Mo-C5	90.80(11)		
C4-Mo-C5	87.47(11)		
C4-Mo-C6	88.14(11)		
C5-Mo-C6	90.52(11)		

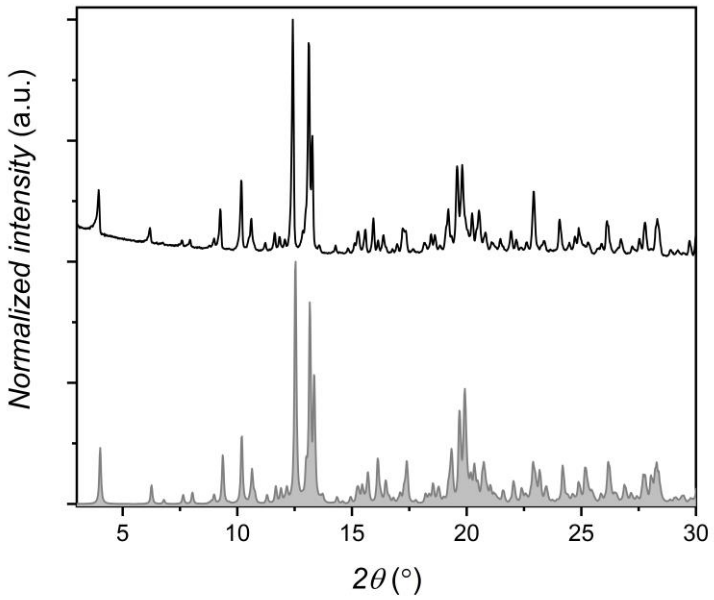

Figure S8. Experimental powder X-ray diffraction pattern recorded for **3** at 296(2) K (black line) and calculated pattern from the single crystal structure studied at 293(2) K (gray line).

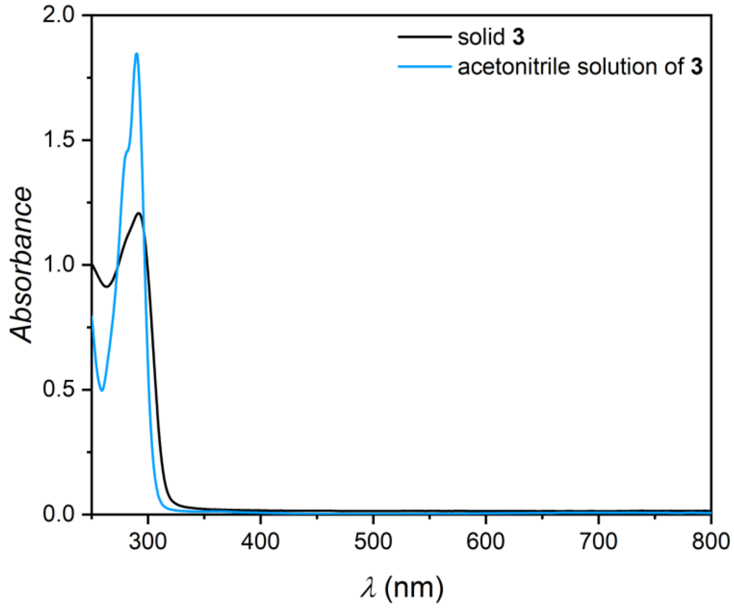

Figure S9. Comparison of the UV-vis spectra of **3** in the solid state (black line) and as a 0.5 mM solution in anhydrous acetonitrile (blue line) at room temperature.

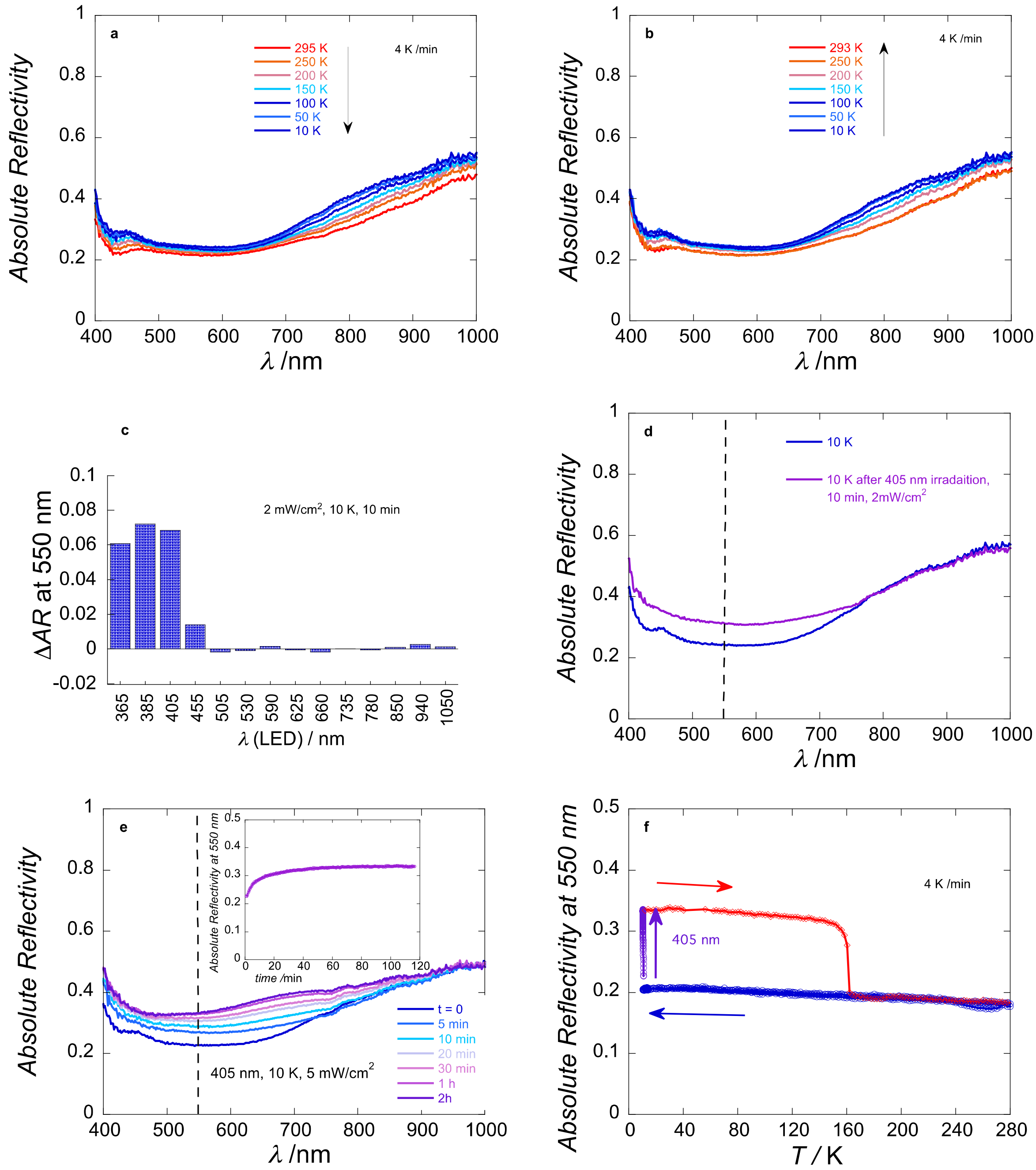

Figure S10. Selected optical reflectivity spectra for **1** between 400 and 1000 nm at selected temperature between 295 and 10 K recorded in the dark and at a scan rate of 4 K min^{-1} in **a**, cooling mode and **b**, heating mode. **c**, Variation of the absolute optical reflectivity (Δ*AR*) recorded at 550 nm for **1** comparing before and after excitation with different LEDs ($\Delta AR = AR_{after} - AR_{before}$; 10 minutes, at 2 mW cm^{-2}) at 10 K (after a fast cooling of the sample from room temperature in the dark). **d**, Optical reflectivity spectra for **1** at 10 K before and after excitation with a 405-nm LED (10 minutes, at 2 mW·cm^{-2}). **e**, Selected optical reflectivity spectra for **1** at 10 K recorded during a 2-hours irradiation with 405-nm LED (at 5 mW·cm^{-2}). In insert, time evolution of the 550-nm *AR* signal during 405-nm irradiation (10 K; 5 mW·cm^{-2}). **f**, Thermal variation of the 550-nm reflectivity signal in the dark (in blue, almost temperature independent around 0.2), during irradiation at 10 K with 405 nm LED (2 mW·cm^{-2}; in violet), and after irradiation in the dark (in red). A spectroscopic white light of 0.08 mW·cm^{-2} was used for these measurements.

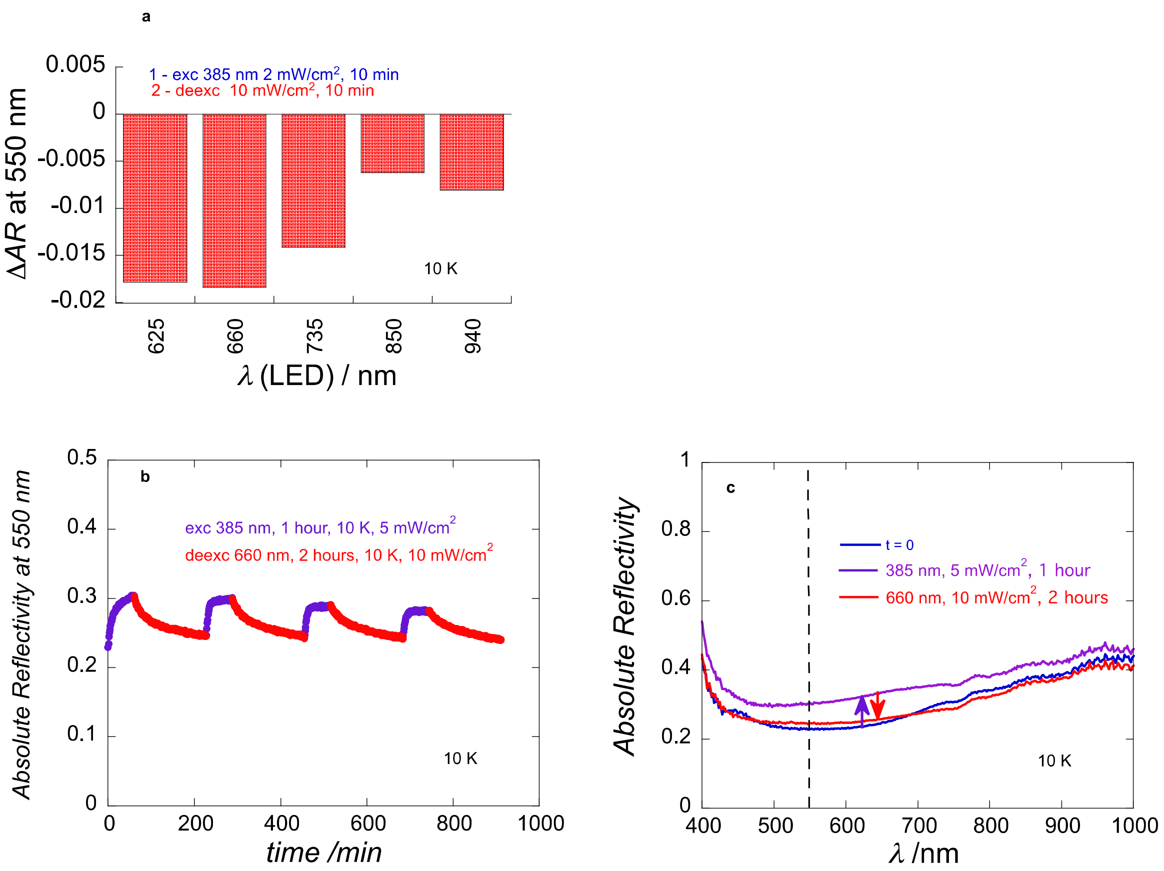

Figure S11. **a**, Variation of the absolute optical reflectivity (Δ*AR*) for **1** recorded at 550 nm and at 10 K (after a fast cooling of the sample from room temperature in the dark and a 385-nm excitation during 10 minutes at 2 mW·cm^{-2}) comparing before and after desexcitation with different LEDs ($\Delta AR = AR_{after} - AR_{before}$; 10 minutes, at 10 mW cm^{-2}). **b**, Time evolution of the 550-nm *AR* signal during four irradiation cycles of 385 nm irradiation (10 K; 1 hour, 5 mW·cm^{-2}; in violet) and 660 nm irradiation (10 K; 2 hours, 10 mW·cm^{-2}; in red). **c**, Selected optical reflectivity spectra for **1** at 10 K recorded in the dark (blue trace), after one 385-nm irradiation (10 K; 1 hour, 5 mW·cm^{-2}; violet trace) and a subsequent 660 nm irradiation (10 K; 2 hours, 10 mW·cm^{-2}; red trace). A spectroscopic white light of 0.08 mW·cm^{-2} was used for these measurements.

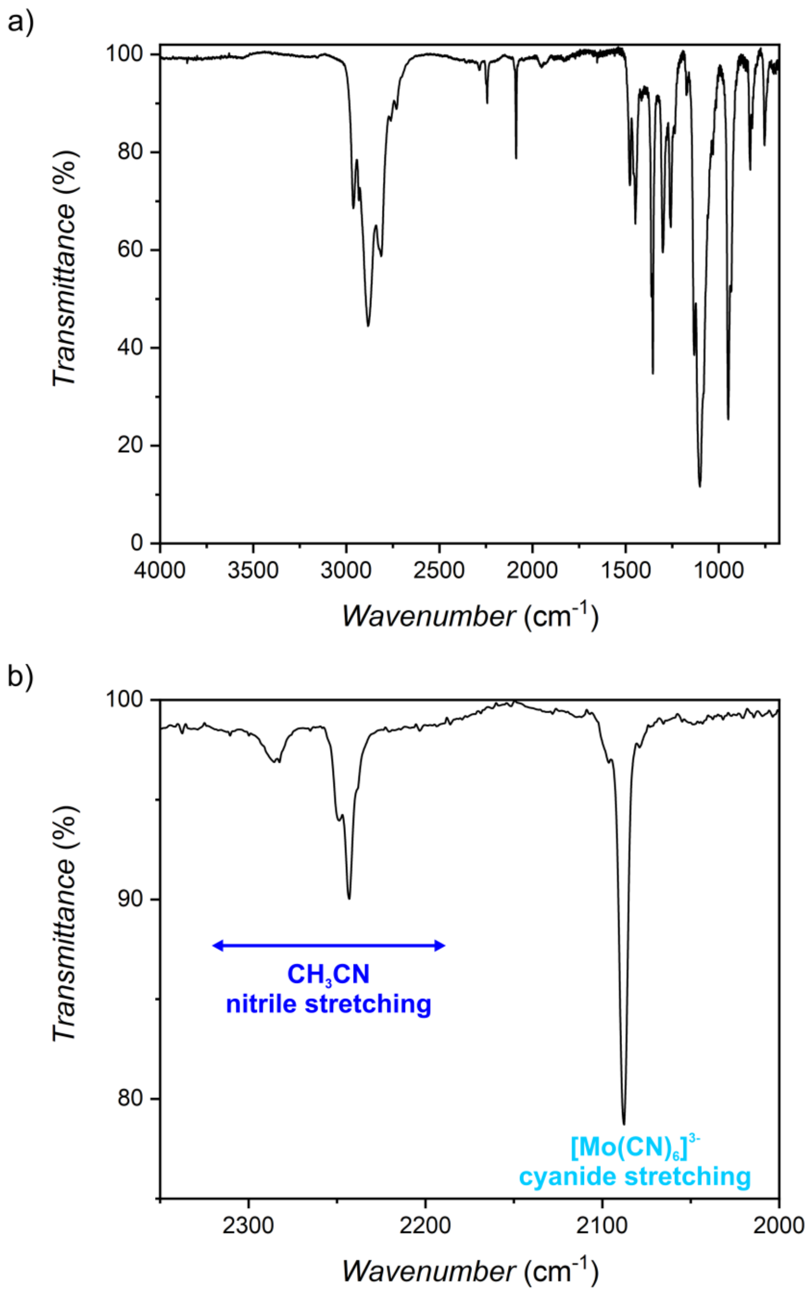

Figure S12. a, IR spectrum of **3** recorded in the solid state at room temperature and **b**, close-up of the cyanide stretching region.

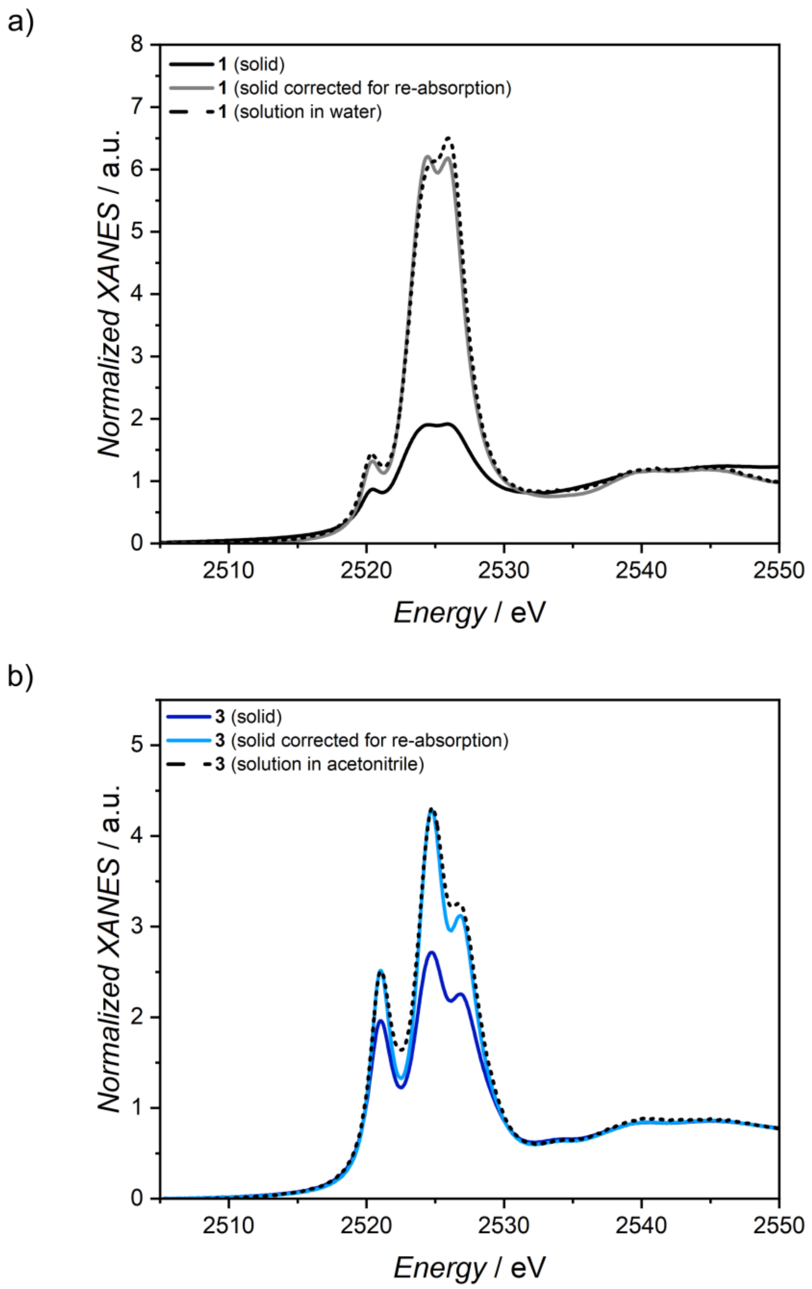

Figure S13. Mo L_3-edge XANES spectra at room temperature of **1** in the solid state (solid line) and in water solution (dashed line) (**a**) and **3** in the solid state (solid line) and in the acetonitrile solution (dashed line).

Table S5. Positions and assignments of all excitations from theoretical calculations using CASSCF/CASPT2 (complete active space self consistent field/complete active space perturbation theory) approaches for metal-centered states of **1**.[a]

State	CASSCF (eV)	CASPT2 (eV)	CASSCF (nm)	CASPT2 (nm)
MC	0.00	0.00	---	---
MC	0.28	0.30	---	---
MC	2.31	2.69	536.7	460.9
MC	2.89	3.32	429.0	373.4
MC	3.23	3.43	383.9	361.5
MC	3.40	3.60	364.7	344.4
MC	3.42	3.61	362.5	343.4
MC	3.71	4.12	334.2	300.9
MC	3.79	4.07	327.1	304.6
MC	3.84	4.08	322.9	303.9
MC	4.14	4.32	299.5	287.0
MC	4.28	4.38	289.7	283.1
MC	4.62	4.84	268.4	256.2
MC	4.70	4.90	263.8	253.0
MC	5.23	5.22	237.1	237.5
MC	5.52	5.42	224.6	228.8
LMCT	5.95	4.79	208.4	258.8
LMCT/MC	6.04	5.52	205.3	224.6
LMCT	6.30	4.63	196.8	267.8
MC	6.45	7.09	192.2	174.9
MC	6.84	7.49	181.3	165.5
MC/π-π*	7.00	6.82	177.1	181.8
MC	7.08	7.72	175.1	160.6
MC	7.13	7.64	173.9	162.3
MC/π-π*	7.26	7.05	170.8	175.9
MC	7.36	7.76	168.5	159.8
MC	7.41	7.95	167.3	156.0
MC	7.45	7.85	166.4	157.9
MC	7.59	8.06	163.4	153.8
MC	7.74	8.29	160.2	149.6
MC	7.85	8.48	157.9	146.2
MC	7.85	8.29	157.9	149.6
MC	7.90	8.33	156.9	148.8

[a]quartet states (S = 3/2) are marked with blue background; MC: metal centered; LMCT: ligand metal charge transfer.

Table S6. Positions and assignments of all excitations from theoretical calculations using CASSCF/CASPT2 approaches for metal-centered states of **2**.[a]

State	CASSCF (eV)	CASPT2 (eV)	CASSCF (nm)	CASPT2 (nm)
$^4A_{2g}$	0	0	---	---
^2E_g (t^3)	1.10	1.29	1127.1	961.1
	1.10	1.30	1127.1	953.7
$^2T_{1g}$ (t^3)	1.15	1.34	1078.1	925.3
	1.16	1.35	1068.8	918.4
	1.17	1.34	1059.7	925.3
$^2T_{2g}$ (t^3)	2.06	2.05	601.9	604.8
	2.09	2.11	593.2	587.6
	2.09	2.11	593.2	587.6
$^4T_{2g}$	4.68	5.07	264.9	244.5
	4.69	5.08	264.4	244.1
	4.72	5.18	262.7	239.4
$^4T_{1g}$	5.45	5.74	227.5	216.0
	5.51	5.80	225.0	213.8
$^2A_{1g}$ (t^2e^1)	5.51	6.05	225.0	204.9
LMCT/π-π*	5.53	4.81	224.2	257.8
other t^2e^1 doublets	5.64	6.19	219.8	200.3
	5.67	6.22	218.7	199.3
	5.68	6.22	218.3	199.3
$^4T_{1g}$	5.76	6.06	215.3	204.6
other t^2e^1 doublets	5.77	6.28	214.9	197.4
	5.78	6.30	214.5	196.8
	5.79	6.29	214.1	197.1
LMCT	5.86	5.17	211.6	239.8
other t^2e^1 doublets	5.93	6.38	209.1	194.3
	5.99	6.42	207.0	193.1
	6.22	6.62	199.3	187.3
	6.25	6.65	198.4	186.4

[a]doublet states are marked with an orange background.

Details of spin-polarized periodic DFT computations

To reproduce the structural transformations from **1** to **2** and back, a computational protocol was developed involving various optimization approaches and density functionals. Three hybrid functionals (PBE0[4, 5] PBE0-13[6], and B3LYP[7-9]) and four range-separated hybrid functionals (LC-PBE,[4, 10, 11] LC-ωPBE,[4, 12] HISS,[13, 14] and CAM-B3LYP[7, 8, 15]) were tested. The D3 dispersion correction with Becke–Johnson damping function was applied for PBE0, B3LYP, and LC-ωPBE.[14, 15] The protocol included two optimization types in CRYSTAL23[16] – full optimization of unit cell parameters and atom positions (FO) and optimization of atom positions within a fixed unit cell (AO). Initial models, based on scXRD data with added hydrogen atoms (positionned at electron density maxima according to chemical criteria), were optimized using AO and FO to simulate spin-state transformations, **1**→**2** and **2**→**1**. FO re-optimization then used a spin-lock to fix the (α-β) electron occupancy matching the target spin state during the first 50 SCF (self consisten field) cycles. Transitions were simulated in both preserved and reduced symmetry ($P\bar{1}$ and P1) to determine if symmetry breaking is part of the transformations. Results were evaluated by comparing experimental and computed structural parameters (e.g., unit cell, Mo···CN distances) and spin density at the Mo centers, estimated according to the atomic charges ($Z_{MUL(\alpha\text{-}\beta)}$) obtained in the Mulliken population analysis of α-β electrons.[17] To test internal consistency, a Figure of Merit (FoM) parameter was defined for calculations using each functional:

$$FoM = \frac{V[\boldsymbol{a}\rightarrow\boldsymbol{b}]-V[\boldsymbol{b}]}{V[\boldsymbol{b}]} \qquad \text{Eq. 1}$$

where the unit cell volumes V[**a**→**b**] and V[**b**] represent V[**1**→**2**] and V[**2**] for a **1**→**2** transformation, and V[**2**→**1**] and V[**1**] for a **2**→**1** transformation. As such, a FoM should be equal to zero if the transformation induced by the spin-lock procedure perfectly replicates the target spin state's optimized geometry. Evaluating these parameters across functionals showed no significant differences between calculations in $P\bar{1}$ and $P1$ symmetry. Additionally, re-optimization from both AO and FO geometries yielded nearly identical results, despite different starting unit cell parameters and coordinates. Consequently, only FO $P\bar{1}$ results are discussed further.

Given that structural models were based on 30 K scXRD measurements, accurate reproduction of unit cell parameters and volume was anticipated for both optimized spin states. Depending on the DFT functional, results showed either contraction or inflation of the unit cells (Table S7). Functionals underestimating volume tended to replicate the smaller unit cell of **1** more accurately, while those overestimating better reproduced V[**2**]. The PBE0-D3 and HISS functionals yielded the closest volumes for **1** and **2**, respectively. For **2**, the Mo···CN distance in the dissociated cyanide correlated well with the optimized unit cell size, with HISS almost perfectly matching the experimental distance (Fig. S14, Table S7). Comparison of $Z_{MUL(\alpha\text{-}\beta)}$ values at the Mo centers (Table S8) highlighted the range-separated functionals as superior in capturing the expected number of unpaired electrons for each spin state.
Each functional successfully replicated the **1**→**2** structural transformation when the spin-lock procedure was applied. In all cases, one cyanide group dissociated from the Mo center early in re-optimization, stabilizing through two nearly linear hydrogen bonds with water molecules. For each functional, unit cell volume, $Z_{MUL(\alpha\text{-}\beta)}$ values at the Mo centers, and the position of the dissociated CN^- resulting from the re-optimization of **1** are almost identical to that obtained after the optimization of **2** starting from the experimental data (Table S7-S8, Fig. S14-S15). However, only PBE-based functionals reproduced the reverse **2**→**1** transformation (Table S8). For B3LYP and CAM-B3LYP, the structure did not shift to a smaller volume, and CN^- association was absent (Table S7, Fig. S15). Overall, the HISS DFT functional provided the best alignment with experimental data (Fig. S16-S17).

Table S7. Relative errors in reproducing experimental unit cell volumes (**1**$_{test}$ for Δ*V*[**1**] and Δ*V*[**2**→**1**], **2** for Δ*V*[**1**→**2**] and Δ*V*[**2**]) for studied DFT functionals.

Method	Δ*V*[1] (%)	Δ*V*[1→2] (%)	Δ*V*[2] (%)	Δ*V*[2→1] (%)
**B3LYP-D3**	-0.95	-3.11	-2.96	5.87*
**CAM-B3LYP**	4.05	1.95	1.96	11.47*
**LC-PBE**	-2.59	-5.06	-5.11	-3.10
**LC-ωPBE-D3**	-2.25	-4.58	-4.56	-2.27
**PBE0-D3**	-0.78	-3.02	-2.99	-0.79
**PBE0-13**	3.96	2.06	2.09	3.94
**HISS**	2.93	0.95	0.96	2.93

*6-coordinate Mo centers

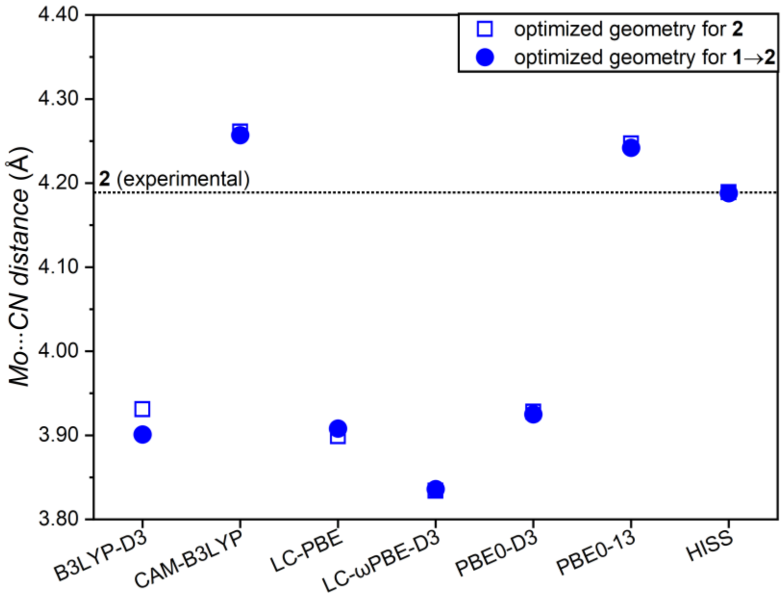

Figure S14. Comparison of Mo···CN distances (for the dissociated CN⁻ ligand) obtained by geometry optimization of structures **2** and **1**→**2** for studied DFT functionals. Please note that in most cases the circles and squares overlap.

Table S8. Comparison of atomic charges at Mo centers ($Z_{MUL(\alpha-\beta)}$ in a.u.) obtained in the Mulliken population analysis of α-β electrons for studied DFT functionals.

Method	$Z_{MUL(\alpha-\beta)}$[1] (a.u.)	$Z_{MUL(\alpha-\beta)}$[1→2] (a.u.)	$Z_{MUL(\alpha-\beta)}$[2] (a.u.)	$Z_{MUL(\alpha-\beta)}$[2→1] (a.u.)
**B3LYP-D3**	0.833	2.710	2.711	0.898*
**CAM-B3LYP**	0.900	2.841	2.841	0.950*
**LC-PBE**	0.920	2.920	2.920	0.935
**LC-ωPBE-D3**	0.923	2.929	2.929	0.923
**PBE0-D3**	0.850	2.774	2.774	0.850
**PBE0-13**	0.893	2.855	2.855	0.893
**HISS**	0.910	2.888	2.888	0.910

*6-coordinate Mo centers

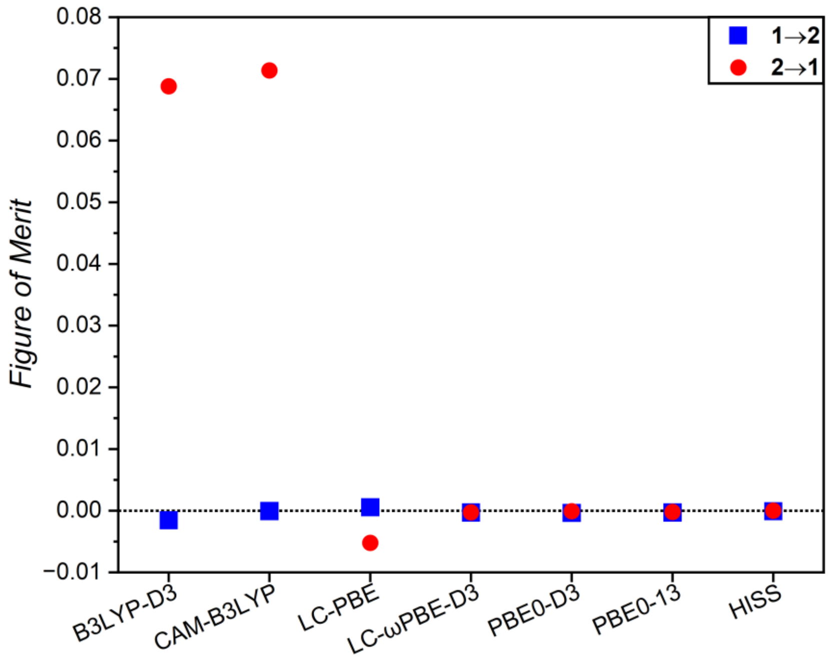

Figure S15. Figure of Merit (defined as described above) obtained by geometry optimization of structures **1→2** and **2→1** for studied DFT functionals.

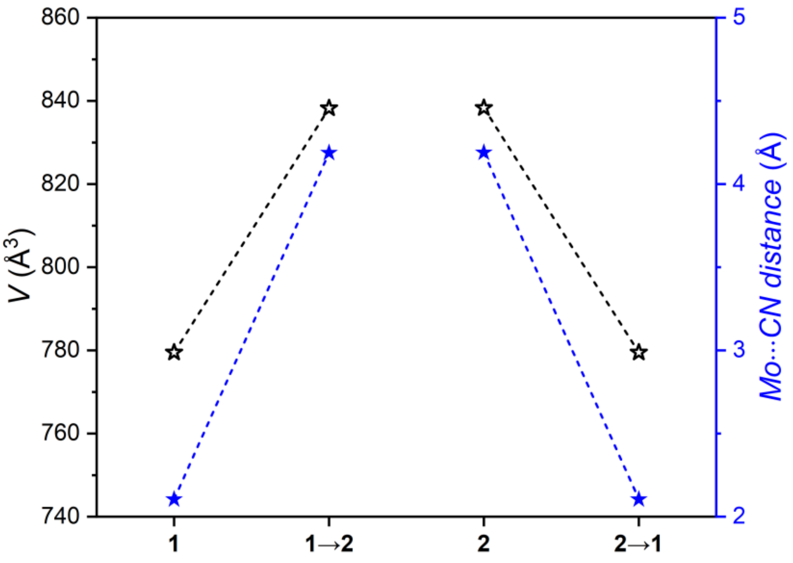

Figure S16. Mo···CN distances for the cyanide ligand that undergoes photodissociation (blue; right axis) and unit cell volumes (black; left axis) for the optimized geometries of **1**, **1→2**, **2** and **2→1** using HISS functional.

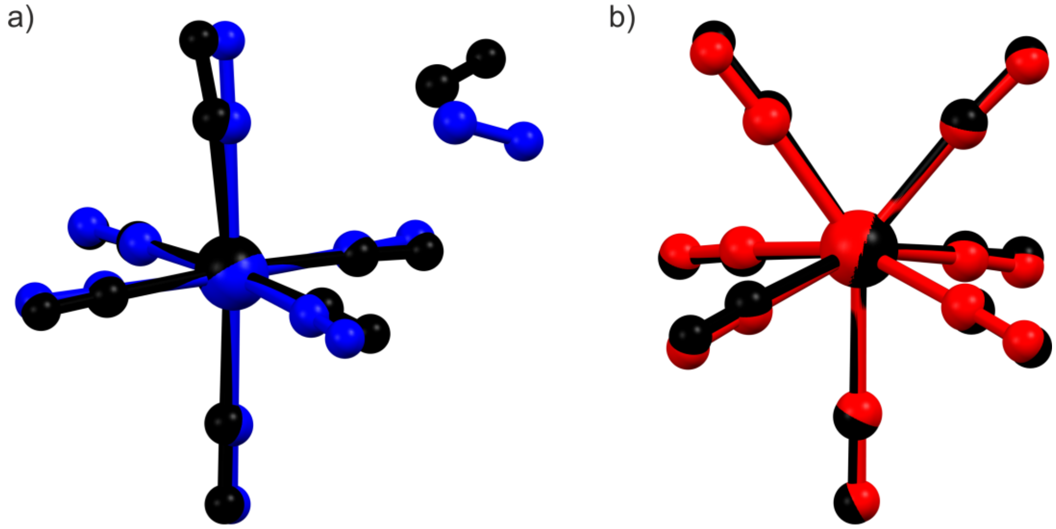

Figure S17. Overlay of the crystal structures: **a**, experimental **2** (black) and optimized **1**⟶**2** (blue) and **b**, experimental **1** (black) and optimized **2**⟶**1** (red), for HISS functional.

Description of the bulk magnetic properties of $K_4[Mo^{III}(CN)_7]\cdot 2H_2O$ (1) in the ground state

The magnetic susceptibility (χ) of **1** was analysed using the Bonner-Fisher model for a uniform chain of antiferromagnetically coupled S = 1/2 Heisenberg spins (Fig. S19a):[18, 19]

$$\hat{H} = -2J\sum_{i=1}^{N}\vec{S}_i\cdot\vec{S}_{i+1} - g\mu_B\sum_{i=1}^{N}\vec{H}\cdot\vec{S}_i \qquad \text{Eq. 2}$$

Assuming S = 1/2 molybdenum centers and utilizing g-factors derived from EPR spectroscopy, the isotropic superexchange coupling was found to be J/k_B = -4.2(1) K (fitting temperature range 2-300 K). This model aligns well with the crystal structure of **1**, where the N1···N7 distance of 3.40(1) Å is the smallest separation among heptacyanomolybdate(III) complexes, facilitating th150
e formation of a supramolecular chain (Fig. S20). Furthermore, intermolecular interactions are mediated by CN···K···NC bridges, supporting the description via a superexchange mechanism. However, the model does not adequately account for the magnetic susceptibility below 15 K, likely due to interchain interactions in a densely packed structure of **1** and the presence of an antiferromagnetically ordered state below 3.5 K (vide infra).

The heat capacity (C_p) measurements reveal a broad maximum at 3.5 K which is lower than the magnetic susceptibility (χ) maximum at 5.5 K (Fig. S19). This is consistent with the low dimensionality of **1** and its supramolecular spin chain arrangement.[18] The magnetic entropy change associated to this C_p peak, after subtracting the phonon contribution (red line in Fig. S19b), is approximately ΔS = 4.9 $J\cdot K^{-1}\cdot mol^{-1}$ in the 0.5-10 K range. This value is only slightly lower than the expected $R\ln 2$ = 5.76 $J\cdot K^{-1}\cdot mol^{-1}$ for an ordered S = ½ system. These correlations reduce the experimentally determined ΔS and contribute to the absence of a sharp lambda-shaped peak in the specific heat at the magnetic transition.

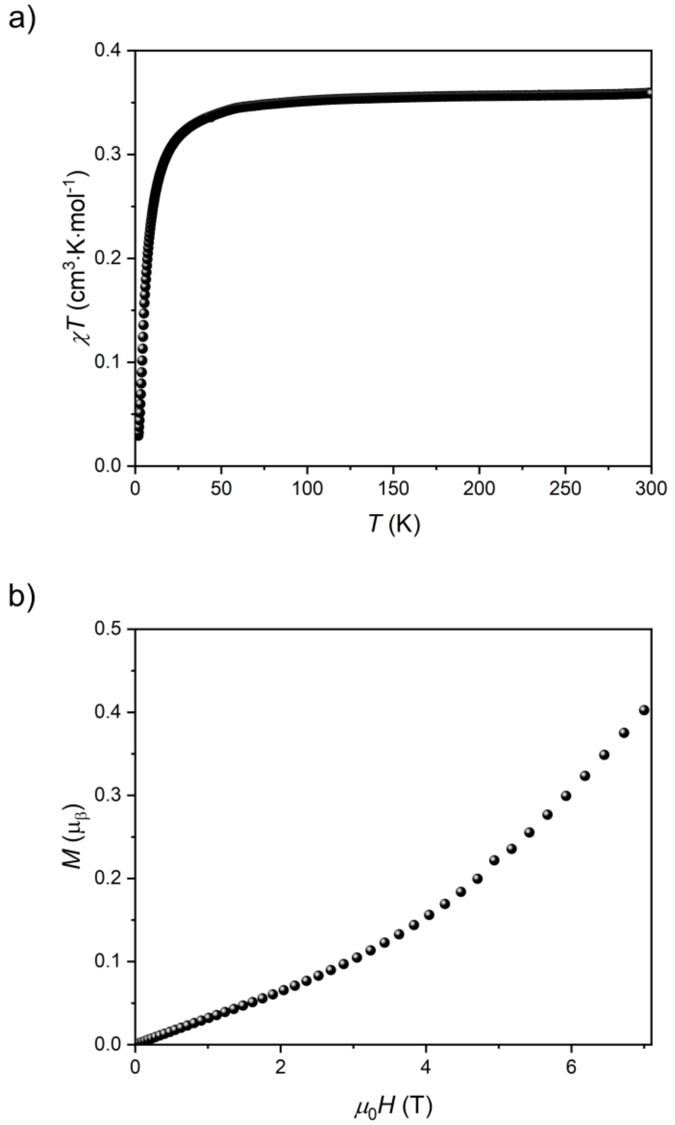

Figure S18. a, Temperature (*T*) dependence of the χT product at 0.1 T for **1** (where χ = *M*/*H* is the molar magnetic susceptibility normalized per complex) and **b**, field (*H*) dependence of the magnetization (*M*) for **1** at 1.8 K.

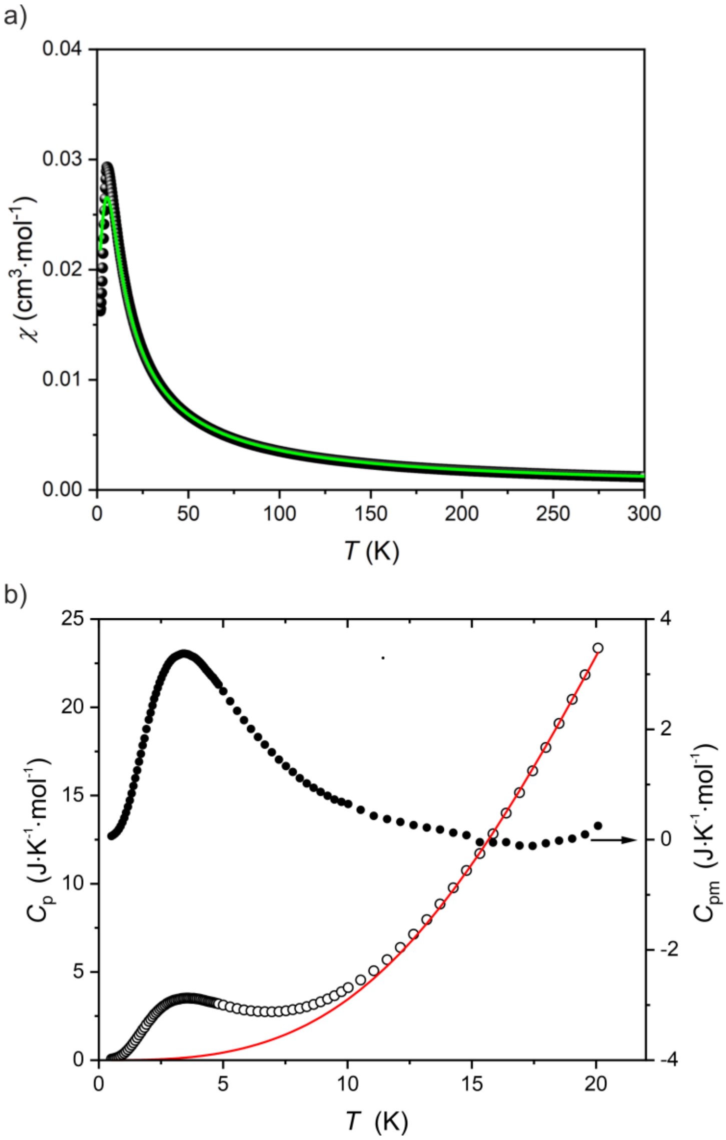

Figure S19. a, Temperature (T) dependence of the molar magnetic susceptibility, χ, at 0.1 T for **1** (black points) and fit to Bonner-Fisher model for a uniform chain of antiferromagnetically coupled S = 1/2 Heisenberg spins (green line; *vide supra*). **b**, Temperature dependence of the heat capacity, $C_p(T)$, for **1** under zero magnetic field (open circles), Debye model used to determine the nonmagnetic phonon contribution to the heat capacity ($C_{background}$, red line), and the magnetic contribution to the heat capacity, C_{pm}, defined as C_p-$C_{background}$ (full circles).

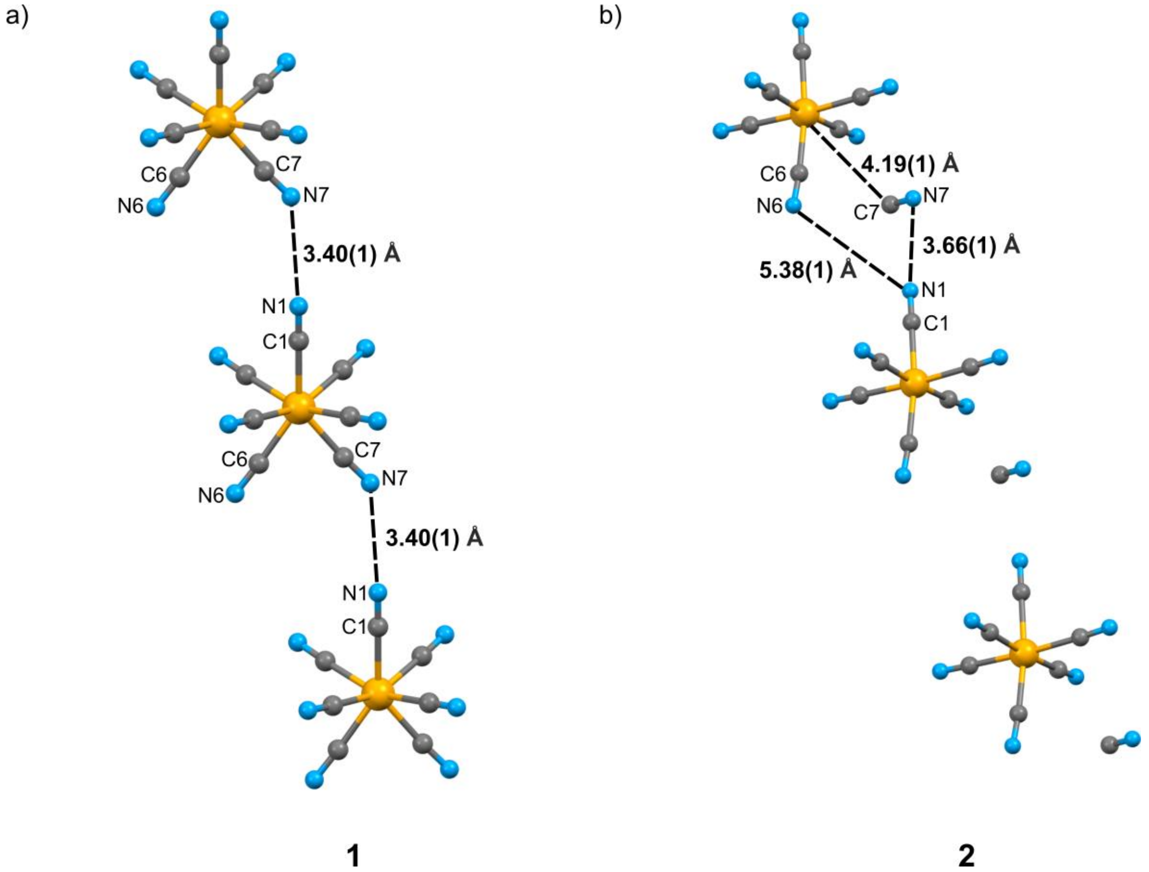

Figure S20. Fragments of the crystal structures at 30 K of **1** (a) and **2** (b) highlighting the supramolecular chain arrangement in **1**, and its disappeareance in **2** due to the cyanide dissociation.

Description of the bulk magnetic properties of [K(crypt-222)]$_3$[Mo^{III}(CN)$_6$]·2CH$_3$CN (3)

The temperature dependence of magnetic susceptibility, χ, and its temperature product (χT) recorded for **3** in the 2-300 K range, were accurately reproduced by a S = 3/2 Curie law with g_{Mo} = 1.97(2) (Fig. S21).[20] Both the spin state of 3/2 and the g-factor slightly below 2.00 are in line with the expected $t_{2g}{}^3e_g{}^0$ electronic configuration for Mo(III) in octahedral geometry. Accordingly, the magnetization value of 2.86 μ_B at 2 K and 7 T closely matches with 2.94 μ_B deduced from the S = 3/2 Brillouin function (Fig. S22).

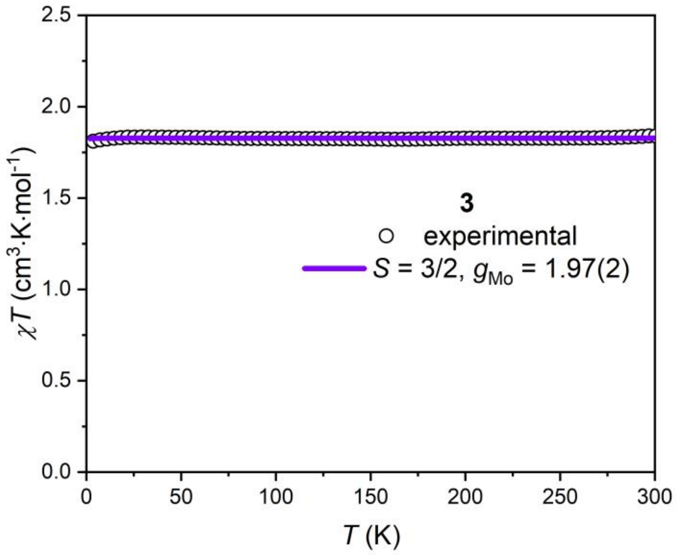

Figure S21. Temperature (T) dependence of the χT product at 0.1 T for **3** (where $\chi = M/H$ is the molar magnetic susceptibility normalized per complex). The solid purple line is the best fit of the data to a S = 3/2 Curie law (with g_{Mo} being the only ajustable parameter).

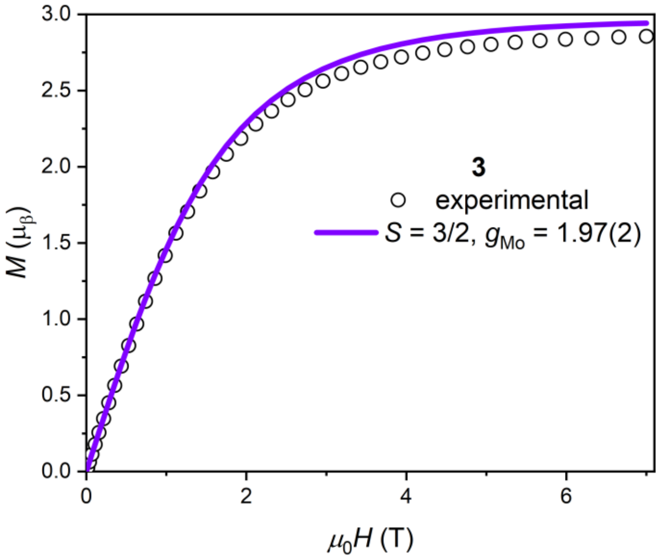

Figure S22. Field (H) dependence of the magnetization (M) for **3** at 2.0 K. The solid purple line is the simulated S = 3/2 Brillouin function with g_{Mo} =1.97.

Table S9. Stretched exponential decay fits of the relaxation data for **2** shown in Fig. 3e.[a]

T (K)	τ (s)	β	$\Delta\chi T$ / cm^3·K·mol^{-1}	χT_0 / cm^3·K·mol^{-1}	R^2
132.5	10757(9)	1.339(2)	1.150(1)	0.4337(4)	0.99893
135	5305(5)	1.300(2)	1.181(1)	0.4321(2)	0.99899
137.5	3040(5)	1.328(4)	1.032(2)	0.4430(5)	0.99898
140	1688(4)	1.327(6)	1.074(2)	0.4399(7)	0.99858
142.5	939(3)	1.363(8)	1.02(3)	0.4410(7)	0.99818
145	491(2)	1.312(10)	0.945(3)	0.4389(6)	0.99820
147.5	260(2)	1.242(16)	0.781(5)	0.4412(9)	0.99710

[a]All data were fitted to the following equation (Eq. 3): $\chi T = \Delta\chi T \cdot e^{-\left(\frac{t}{\tau}\right)^{\beta}} + \chi T_0$

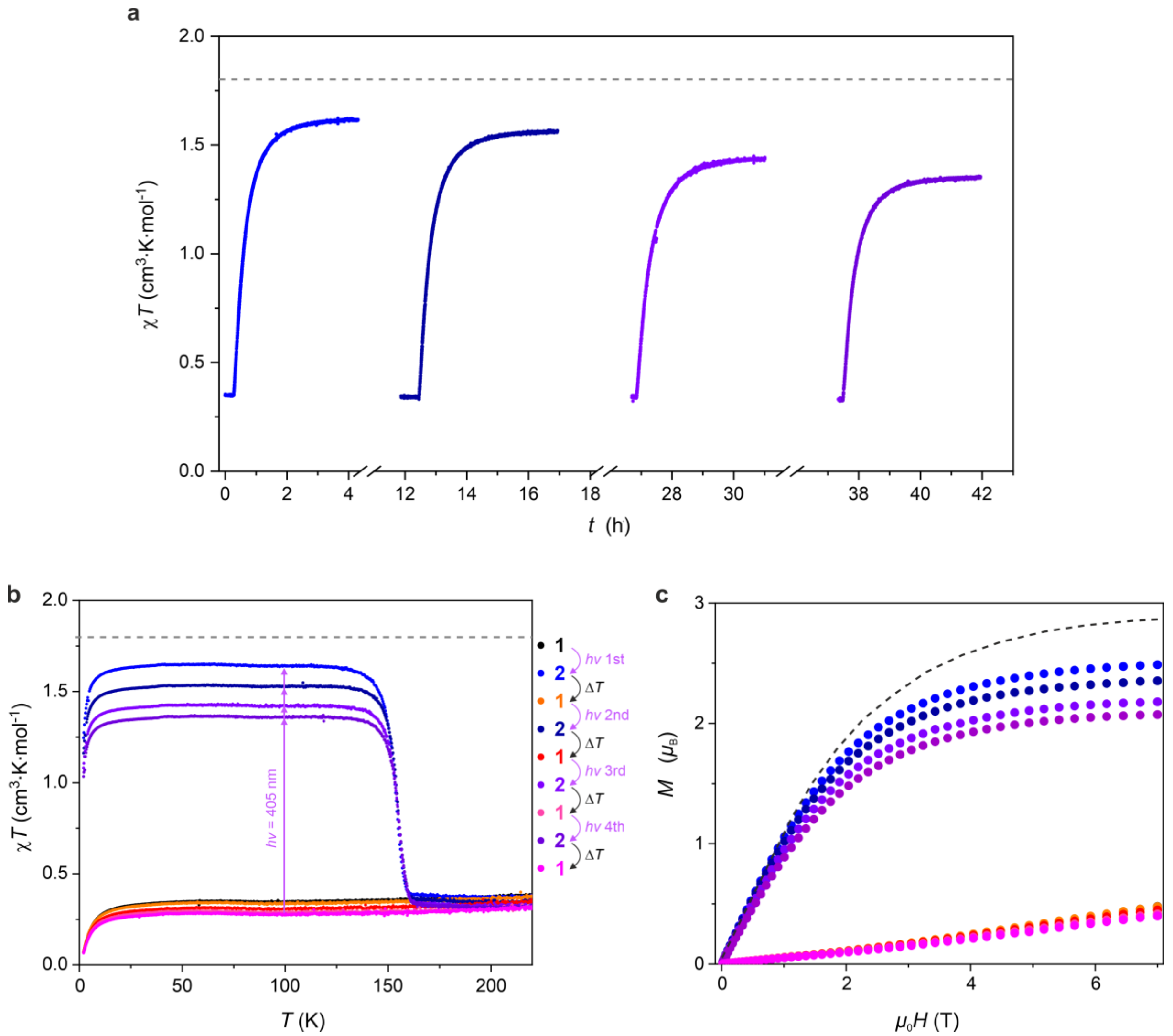

Figure S23. Photomagnetic studies of 1 after irradiation at 100 K. a, Time evolution of the χT product (at 0.1 T) of **1** during 4 consecutive cycles of 405 nm irradiation at 100 K, each followed by thermal relaxation at 220 K, confirming reversible photo-induced transformation **1**⟶**2**; **b**, Temperature dependence of the χT product at 0.1 T before (**1**) and after 405 nm irradiation (**2**) at 100 K in four consecutive irradiation and heating cycles as shown in Fig. S22a; **c**, Magnetization (*M*) versus magnetic field (*H*) plots for **1** (before irradiation) and **2** (after 405 nm irradiation) recorded at 2.0 K after four consecutive irradiation and heating cycles as shown in Fig. S22a. Dashed lines in all three plots indicate the χT (**a** and **b**) or M (**c**) values reached after the analogous irradiation experiments performed at 10 K presented in Figure 3.

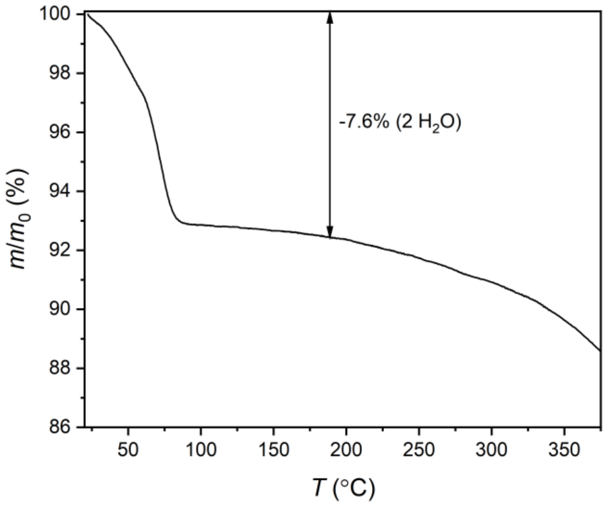

Figure S24. Thermogravimetric (TG) analysis of $K_4[Mo^{III}(CN)_7]\cdot 2H_2O$ (**1**) showing the complete loss of two water molecules of crystallization at 80 °C in a single step corresponding to 7.6% of the formula weight of **1** (*FW* = 470.5 g·mol^{-1}). Data collected under dry N_2 flow with 2 K·min^{-1} heating rate.

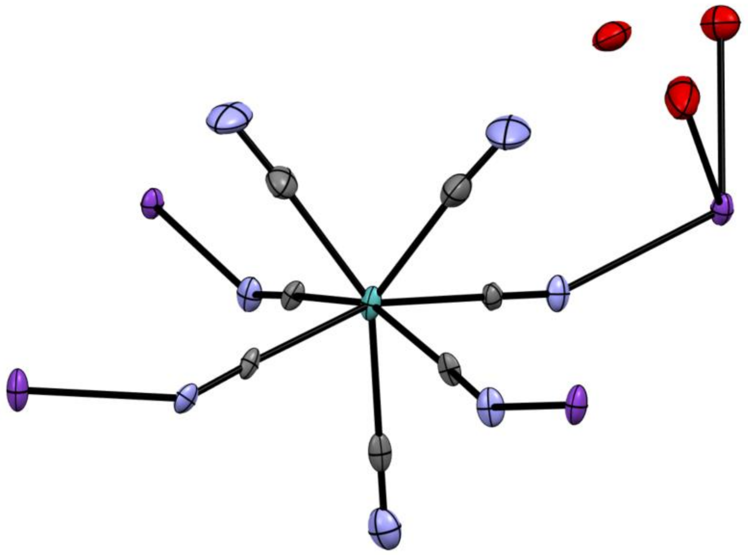

Figure S25. ORTEP-style illustration of the asymmetric unit of **1** (CCDC no. 2352257). Mo – green, K – violet, C – gray, N – blue, O – red. Ellipsoids are depicted at 50% probability level.

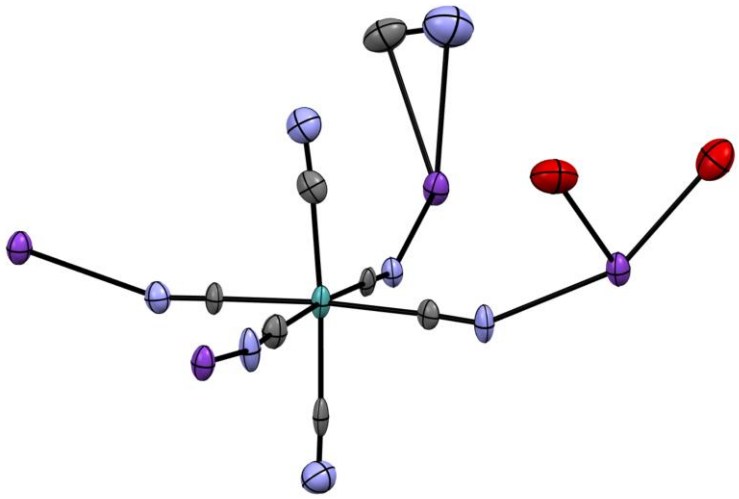

Figure S26. ORTEP-style illustration of the asymmetric unit of **2** (CCDC no. 2352263). Mo – green, K – violet, C – gray, N – blue, O – red. Ellipsoids are depicted at 50% probability level.

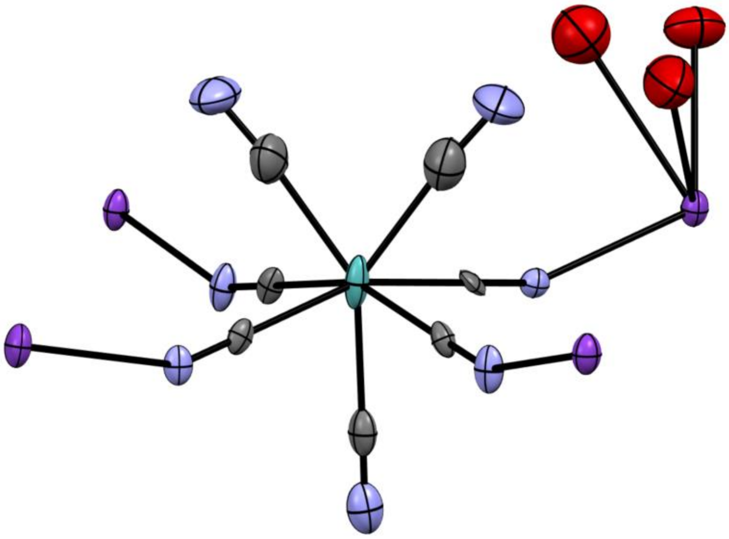

Figure S27. ORTEP-style illustration of the asymmetric unit of **1** relaxed by 638 nm irradiation (CCDC no. 2352259). Mo – green, K – violet, C – gray, N – blue, O – red. Ellipsoids are depicted at 50% probability level.

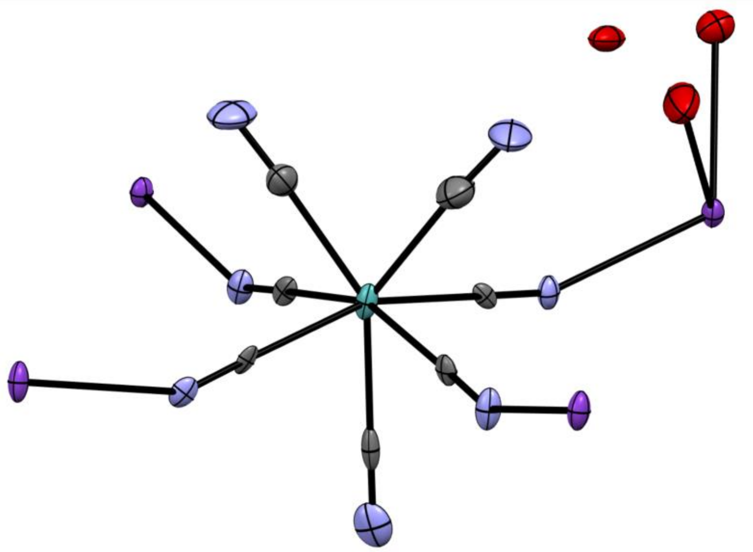

Figure S28. ORTEP-style illustration of the asymmetric unit of **1’** (CCDC no. 2352264). Mo – green, K – violet, C – gray, N – blue, O – red. Ellipsoids are depicted at 50% probability level.

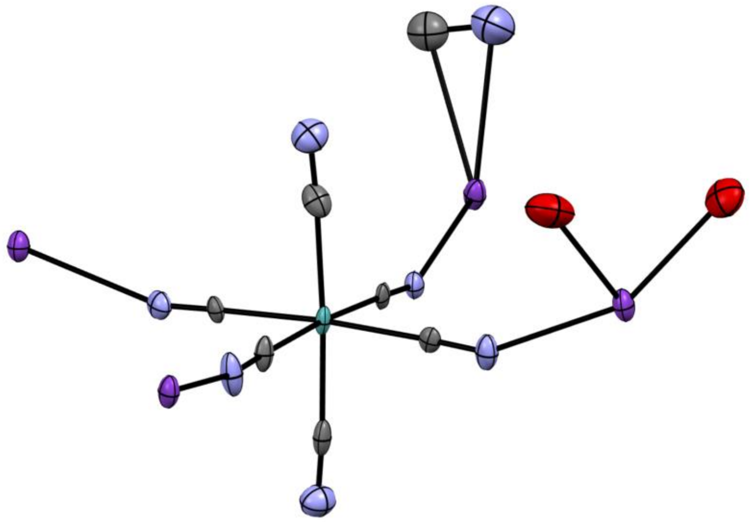

Figure S29. ORTEP-style illustration of the asymmetric unit of **2’** (CCDC no. 2352261). Mo – green, K – violet, C – gray, N – blue, O – red. Ellipsoids are depicted at 50% probability level.

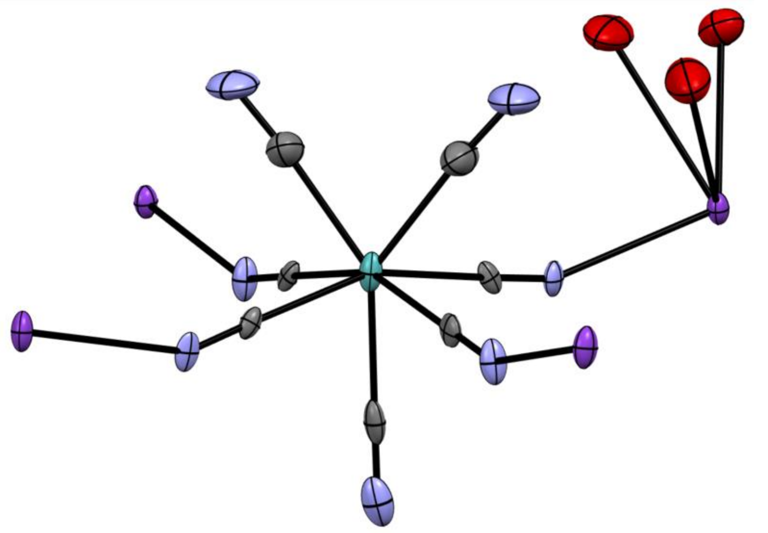

Figure S30. ORTEP-style illustration of the asymmetric unit of **1’** relaxed by heating to 200 K (CCDC no. 2352262). Mo – green, K – violet, C – gray, N – blue, O – red. Ellipsoids are depicted at 50% probability level.

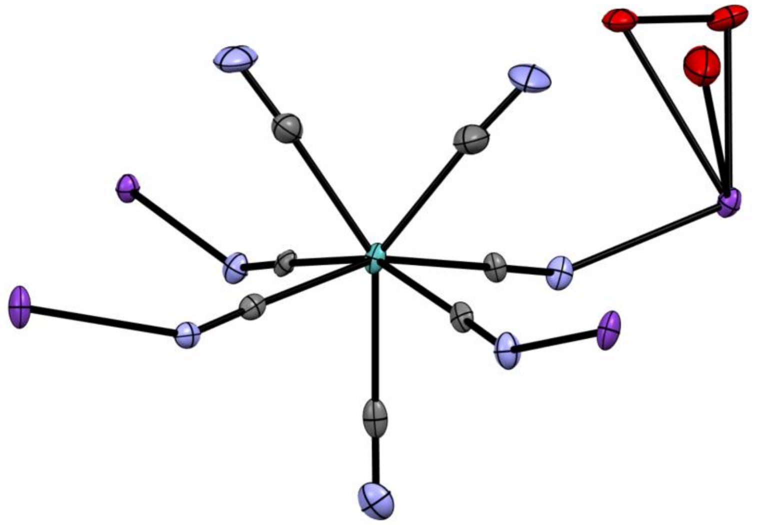

Figure S31. ORTEP-style illustration of the asymmetric unit of $\mathbf{1}_{\mathbf{test}}$ (CCDC no. 2352258). Mo – green, K – violet, C – gray, N – blue, O – red. Ellipsoids are depicted at 50% probability level.

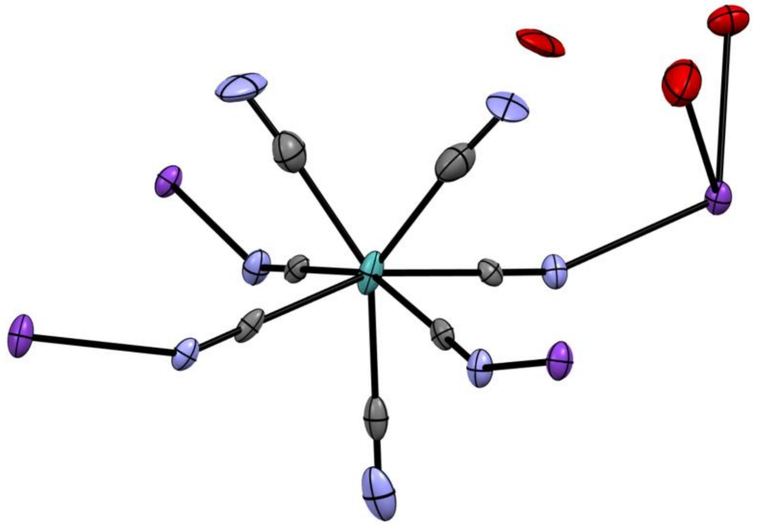

Figure S32. ORTEP-style illustration of the asymmetric unit of $\mathbf{1}_{\mathbf{test2}}$ (CCDC no. 2352260). Mo – green, K – violet, C – gray, N – blue, O – red. Ellipsoids are depicted at 50% probability level.

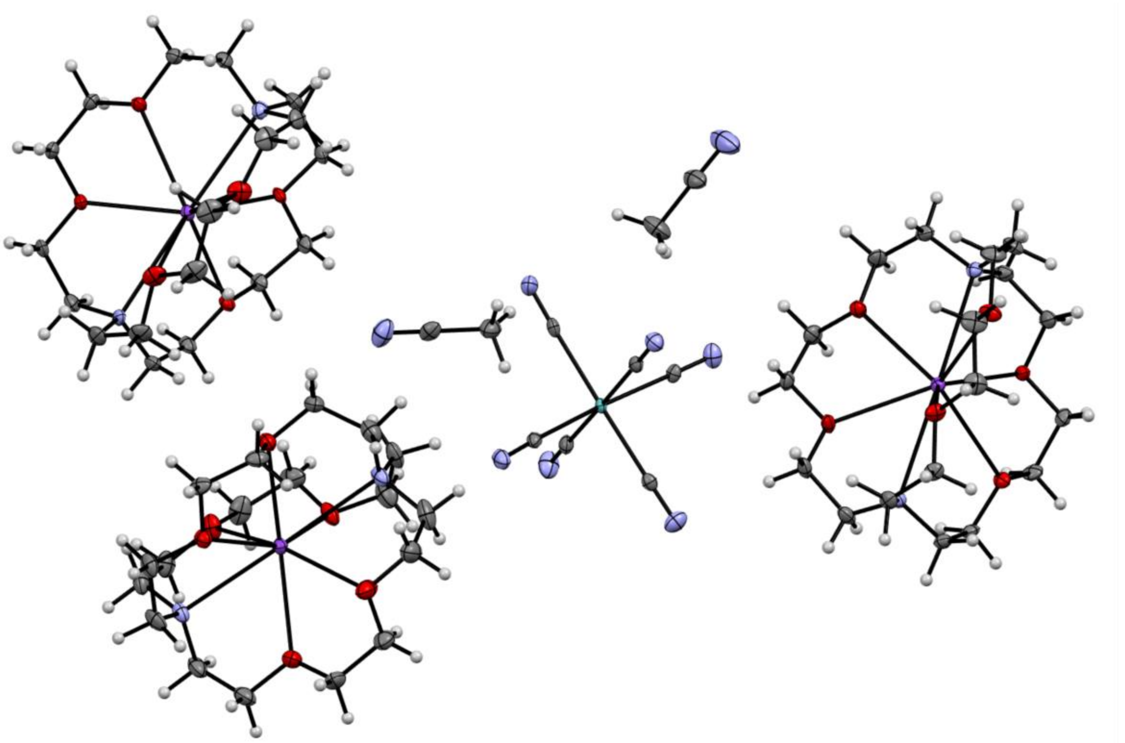

Figure S33. ORTEP-style illustration of the asymmetric unit of **3** at 100 K (CCDC no. 2352265). Mo – green, K – violet, C – gray, N – blue, O – red, H – white. Ellipsoids are depicted at 50% probability level.

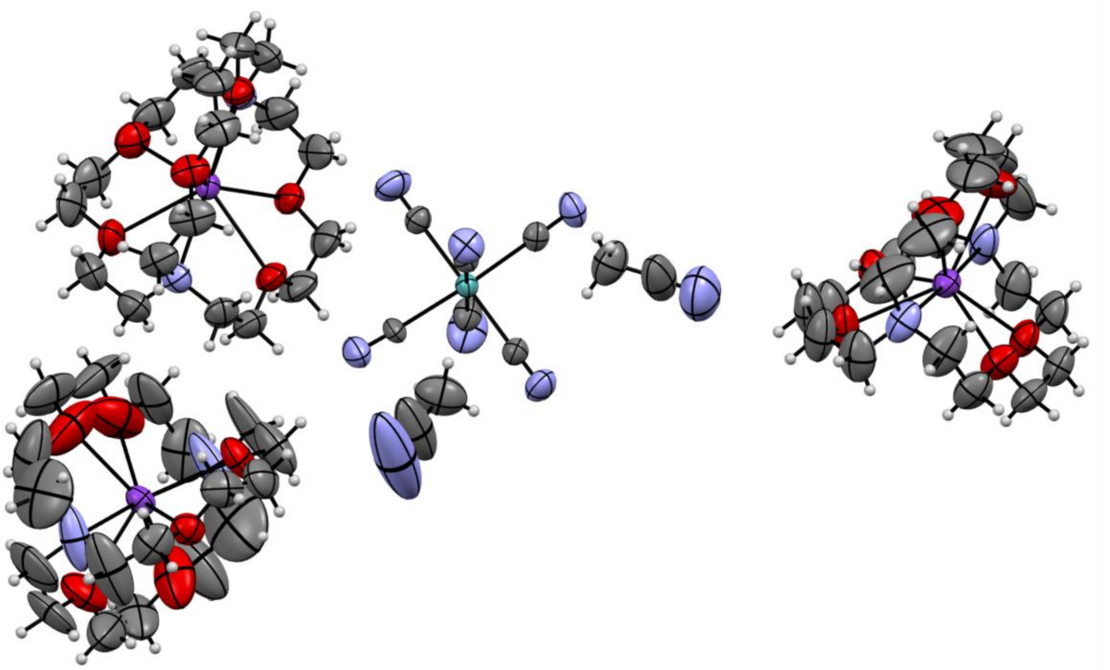

Figure S34. ORTEP-style illustration of the asymmetric unit of **3** at 293 K (CCDC no. 2352266). Mo – green, K – violet, C – gray, N – blue, O – red, H – white. Ellipsoids are depicted at 50% probability level.

Note S1. List of all CheckCif A- and B-level alerts in the reported crystal structures with justifications (the same justifications were included in the cif files deposited with CSD).

1. Crystal structure of **1** (CCDC no. 2352257):

```
PLAT971_ALERT_2_A Check Calcd Resid. Dens. 0.81Ang From Mo1A 4.51 eA-3
PLAT097_ALERT_2_B Large Reported Max. (Positive) Residual Density 4.81 eA-3
```

The large residual density (near molybdenum) is due to partial photoinduced changes in the crystal structure of **1** caused by a strong synchrotron X-ray source.

```
PLAT306_ALERT_2_B Isolated Oxygen Atom (H-atoms Missing ?) ....... O1A Check
```

The water molecules in the structure are disordered, so we decided not to model the positions of the hydrogen atoms, since the refinement led to unphysical results.

2. Crystal structure of **2** (CCDC no. 2352263)

```
LAT306_ALERT_2_B Isolated Oxygen Atom (H-atoms Missing ?) ....... O1B Check
PLAT306_ALERT_2_B Isolated Oxygen Atom (H-atoms Missing ?) ....... O2B Check
```

An attempt to model the position of hydrogen atoms for the photoinduced state led to the instability of the refinement.

3. Crystal structure of **1** relaxed by 638 nm irradiation (CCDC no. 2352259)

```
PLAT971_ALERT_2_A Check Calcd Resid. Dens. 0.91Ang From Mo1A 6.56 eA-3
PLAT097_ALERT_2_B Large Reported Max. (Positive) Residual Density 8.24 eA-3
PLAT971_ALERT_2_B Check Calcd Resid. Dens. 0.78Ang From K4A 2.70 eA-3
PLAT971_ALERT_2_B Check Calcd Resid. Dens. 0.86Ang From K3A 2.61 eA-3
```

LAT973_ALERT_2_B Check Calcd Positive Resid. Density on Mo1A 1.63 eA-3

This is due to slight fatigue of the crystal caused by UV and then red light irradiation.

PLAT306_ALERT_2_B Isolated Oxygen Atom (H-atoms Missing ?) .... O1A Check

The water molecules in the structure are disordered, so we decided not to model the positions of the hydrogen atoms, since the refinement led to unphysical results.

4. Crystal structure of **1’** (CCDC no. 2352264)

PLAT971_ALERT_2_A Check Calcd Resid. Dens. 0.83Ang From Mo1A 5.17 eA-3
PLAT097_ALERT_2_B Large Reported Max. (Positive) Residual Density 5.13 eA-3

The large residual density (near molybdenum) is due to partial photoinduced changes in the crystal structure of **1** caused by a strong synchrotron X-ray source.

PLAT306_ALERT_2_B Isolated Oxygen Atom (H-atoms Missing ?) .... O1A Check

The water molecules in the structure are disordered, so we decided not to model the positions of the hydrogen atoms, since the refinement led to unphysical results.

5. Crystal structure of **2’** (CCDC no. 2352261)

PLAT306_ALERT_2_B Isolated Oxygen Atom (H-atoms Missing ?) .... O1B Check
PLAT306_ALERT_2_B Isolated Oxygen Atom (H-atoms Missing ?) .... O2B Check

An attempt to model the position of hydrogen atoms for the photoinduce state led to instability of the refinement.

PLAT971_ALERT_2_B Check Calcd Resid. Dens. 1.45Ang From C3B 3.47 eA-3
PLAT971_ALERT_2_B Check Calcd Resid. Dens. 0.92Ang From Mo1B 2.58 eA-3
PLAT971_ALERT_2_B Check Calcd Resid. Dens. 1.38Ang From C5B 2.51 eA-3

This is due to slight fatigue of the crystal caused by UV and then red light irradiation.

6. Crystal structure of **1’** relaxed by heating to 200 K (CCDC no. 2352262)

PLAT971_ALERT_2_A Check Calcd Resid. Dens. 0.87Ang From Mo1A 5.92 eA-3
PLAT094_ALERT_2_B Ratio of Maximum / Minimum Residual Density .... 6.48 Report
PLAT097_ALERT_2_B Large Reported Max. (Positive) Residual Density 6.74 eA-3
PLAT973_ALERT_2_B Check Calcd Positive Resid. Density on Mo1A 1.62 eA-3

This is due to slight fatigue of the crystal caused by UV light radiation followed by thermal treatment.

PLAT306_ALERT_2_B Isolated Oxygen Atom (H-atoms Missing ?) .... O1A Check

The water molecules in the structure are disordered, so we decided not to model the positions of the hydrogen atoms, since the refinement led to unphysical results.

7. Crystal structure of $\mathbf{1_{test}}$ (CCDC no. 2352258)

PLAT971_ALERT_2_A Check Calcd Resid. Dens. 0.79Ang From Mo1A 3.57 eA-3

The large residual density (near molybdenum) is due to partial photoinduced changes in the crystal structure of 1 caused by a strong synchrotron X-ray source.

PLAT306_ALERT_2_B Isolated Oxygen Atom (H-atoms Missing ?) .... O1A Check

The water molecules in the structure are disordered, so we decided not to model the positions of the hydrogen atoms, since the refinement led to unphysical results.

8. Crystal structure of $\mathbf{1}_{\mathbf{test2}}$ (CCDC no. 2352260)

```
PLAT971_ALERT_2_A Check Calcd Resid. Dens. 0.83Ang From Mo1A 6.12 eA-3
PLAT097_ALERT_2_B Large Reported Max. (Positive) Residual Density 6.20 eA-3
```

The large residual density (near molybdenum) is due to partial photoinduced changes in the crystal structure of 1 caused by a strong synchrotron X-ray source.

```
PLAT213_ALERT_2_B Atom N7A has ADP max/min Ratio ..... 5.0 prolat
```

This is due to the onset of cyanide dissociation induced by powerful synchrotron X-ray source.

```
PLAT306_ALERT_2_B Isolated Oxygen Atom (H-atoms Missing ?) ....... O1A Check
```

The water molecules in the structure are disordered, so we decided not to model the positions of the hydrogen atoms, since the refinement led to unphysical results.

9. Crystal structure of **3** at 293 K (CCDC no. 2352266)

```
PLAT230_ALERT_2_B Hirshfeld Test Diff for O9 --C38 . 10.3 s.u.
PLAT230_ALERT_2_B Hirshfeld Test Diff for O10 --C40 . 12.1 s.u.
PLAT230_ALERT_2_B Hirshfeld Test Diff for O10 --C41 . 13.5 s.u.
PLAT230_ALERT_2_B Hirshfeld Test Diff for C33 --C34 . 7.4 s.u.
PLAT230_ALERT_2_B Hirshfeld Test Diff for C35 --C36 . 7.3 s.u.
PLAT230_ALERT_2_B Hirshfeld Test Diff for C37 --C38 . 10.0 s.u.
PLAT241_ALERT_2_B High 'MainMol' Ueq as Compared to Neighbors of O9 Check
PLAT241_ALERT_2_B High 'MainMol' Ueq as Compared to Neighbors of O10 Check
PLAT241_ALERT_2_B High 'MainMol' Ueq as Compared to Neighbors of C25 Check
PLAT241_ALERT_2_B High 'MainMol' Ueq as Compared to Neighbors of C36 Check
PLAT241_ALERT_2_B High 'MainMol' Ueq as Compared to Neighbors of C37 Check
PLAT242_ALERT_2_B Low 'MainMol' Ueq as Compared to Neighbors of K2 Check
PLAT410_ALERT_2_B Short Intra H...H Contact H02S ..H35A . 1.87 Ang.
x,y,z = 1_555 Check
PLAT410_ALERT_2_B Short Intra H...H Contact H02I ..H037 . 1.86 Ang.
x,y,z = 1_555 Check
PLAT410_ALERT_2_B Short Intra H...H Contact H31A ..H38A . 1.86 Ang.
x,y,z = 1_555 Check
```

All these alerts are due to the data collection performed at room temperature at which the crystal begins to lose its crystallization solvent resulting in a deterioration of structural parameters. Please note that diffraction experiment at 100 K (CCDC no. 2352265) was performed for the same crystal leading to no A- or B-levele alerts.

```
PLAT910_ALERT_3_B Missing # of FCF Reflection(s) Below Theta(Min). 11 Note
2 0 0, 0 2 0, 1 1 1, 0 2 1, 0 0 2, 1 0 2,
1 1 2, 0 2 2, 1 1 3, 0 0 4, 1 0 4,
```

The diffraction experiment at 293 K was performed using a simplified strategy to collect complete data up to relatively high 2θ angle with some low-anlge reflection obstructed by the beamstop or rejected to to overloads. Please note that diffraction experiment at 100 K (CCDC no. 2352265) was performed for the same crystal leading to no A- or B-levele alerts.

Supplementary References

1. Nakabayashi, K., Tomono, K., Tsunobuchi, Y., Kosaka, W. & Ohkoshi, S.-i. Tetrapotassium heptacyanidomolybdate(III) dihydrate. *Acta Crystallogr. E* **65**, i79-i80 (2009).
2. Alemany, P., Casanova, D., Alvarez, S., Dryzun, C. & Avnir, D. in Reviews in Computational Chemistry 289-352 (2017).
3. Beauvais, L.G. & Long, J.R. Cyanide-Limited Complexation of Molybdenum(III): Synthesis of Octahedral $[Mo(CN)_6]^{3-}$ and Cyano-Bridged $[Mo_2(CN)_{11}]$. *Journal of the American Chemical Society* **124**, 2110-2111 (2002).
4. Perdew, J.P., Burke, K. & Ernzerhof, M. Generalized Gradient Approximation Made Simple. *Phys. Rev. Lett.* **77**, 3865-3868 (1996).
5. Adamo, C. & Barone, V. Toward reliable density functional methods without adjustable parameters: The PBE0 model. *J. Chem. Phys.* **110**, 6158-6170 (1999).
6. Iikura, H., Tsuneda, T., Yanai, T. & Hirao, K. A long-range correction scheme for generalized-gradient-approximation exchange functionals. *J. Chem. Phys.* **115**, 3540-3544 (2001).
7. Lange, A.W., Rohrdanz, M.A. & Herbert, J.M. Charge-Transfer Excited States in a π-Stacked Adenine Dimer, As Predicted Using Long-Range-Corrected Time-Dependent Density Functional Theory. *The Journal of Physical Chemistry B* **112**, 7345-7345 (2008).
8. Weintraub, E., Henderson, T.M. & Scuseria, G.E. Long-Range-Corrected Hybrids Based on a New Model Exchange Hole. *Journal of Chemical Theory and Computation* **5**, 754-762 (2009).
9. Henderson, T.M., Izmaylov, A.F., Scuseria, G.E. & Savin, A. The importance of middle-range Hartree-Fock-type exchange for hybrid density functionals. *J. Chem. Phys.* **127** (2007).
10. Henderson, T.M., Izmaylov, A.F., Scuseria, G.E. & Savin, A. Assessment of a Middle-Range Hybrid Functional. *Journal of Chemical Theory and Computation* **4**, 1254-1262 (2008).
11. Vosko, S.H., Wilk, L. & Nusair, M. Accurate spin-dependent electron liquid correlation energies for local spin density calculations: a critical analysis. *Canadian Journal of Physics* **58**, 1200-1211 (1980).
12. Becke, A.D. Density-functional exchange-energy approximation with correct asymptotic behavior. *Physical Review A* **38**, 3098-3100 (1988).
13. Yanai, T., Tew, D.P. & Handy, N.C. A new hybrid exchange–correlation functional using the Coulomb-attenuating method (CAM-B3LYP). *Chem. Phys. Lett.* **393**, 51-57 (2004).
14. Grimme, S., Antony, J., Ehrlich, S. & Krieg, H. A consistent and accurate ab initio parametrization of density functional dispersion correction (DFT-D) for the 94 elements H-Pu. *J. Chem. Phys.* **132** (2010).
15. Grimme, S., Ehrlich, S. & Goerigk, L. Effect of the damping function in dispersion corrected density functional theory. *J. Comput. Chem.* **32**, 1456-1465 (2011).
16. Erba, A. et al. CRYSTAL23: A Program for Computational Solid State Physics and Chemistry. *Journal of Chemical Theory and Computation* **19**, 6891-6932 (2023).
17. Mulliken, R.S. Electronic Population Analysis on LCAO–MO Molecular Wave Functions. I. *J. Chem. Phys.* **23**, 1833-1840 (1955).
18. Bonner, J.C. & Fisher, M.E. Linear Magnetic Chains with Anisotropic Coupling. *Phys. Rev.* **135**, A640-A658 (1964).
19. Estes, W.E., Gavel, D.P., Hatfield, W.E. & Hodgson, D.J. Magnetic and structural characterization of dibromo- and dichlorobis(thiazole)copper(II). *Inorg. Chem.* **17**, 1415-1421 (1978).
20. Chilton, N.F., Anderson, R.P., Turner, L.D., Soncini, A. & Murray, K.S. PHI: A powerful new program for the analysis of anisotropic monomeric and exchange-coupled polynuclear d- and f-block complexes. *J. Comput. Chem.* **34**, 1164-1175 (2013).
