# Supplementary material for: Reversible single crystal photochemistry and spin state switching in a metal-cyanide complex
Source: Nat Commun. 2025 Sep 29;16:8377. doi: 10.1038/s41467-025-63523-x (PMC12480581; doi:10.1038/s41467-025-63523-x)
Supplement: Supplementary file 2 — Description of Additional Supplementary Files [file 41467_2025_63523_MOESM2_ESM.pdf]

Description of Additional Supplementary Files

File name: Supplementary Movie 1
Description: Animation demonstrating the photodissociation of heptacyanomolybdate(III) anion upon violet light irradiation resulting in hexacyanomolybdate(III) followed by photoassociation induced by red light restoring heptacyanomolybdate(III).

File name: Supplementary Movie 2
Description: Movie showing the change of the color of a single crystal of $K_4[Mo^{III}(CN)_6]\cdot 2H_2O$ upon violet light irradiation at 10 K.
